# Effects of Fe Incorporation Strategies on the Coupled Catalytic and Transport Properties of Fe-Embedded Carbon Molecular Sieve Membranes

**DOI:** 10.1021/acsmaterialsau.6c00171

**Published:** 2026-10-07

**Authors:** Nhan H. Khuu, Mihir Kulkarni, Majed Alam Abir, Antonio Aguirre, Trenton Rosenthal, Alyssa Mize, Joseph ZM Harrah, Madelyn R. Ball, Oishi Sanyal

**Affiliations:** † Department of Chemical and Biomedical Engineering, 5631 West Virginia University, 1306 Evansdale Drive, Morgantown, West Virginia 26506, United States; ‡ Department of Chemical Engineering and Materials Science, 3078 Michigan State University, 428 South Shaw Lane, East Lansing, Michigan 48824, United States

**Keywords:** metal-embedded carbon molecular sieve membrane, catalytic membrane reactor, ammonia synthesis, structure-sensitivity, intrinsic activity, iron-catalyzed reactions

## Abstract

Catalytic membrane reactors offer a promising route to intensify chemical processes by integrating the reaction and selective product removal in a single unit. Carbon molecular sieve (CMS) membranes are attractive candidates for membrane reactors because of their strong molecular sieving capability, high thermal and pressure stability, and the presence of functional groups that enable metal incorporation. Although metal-containing CMS membranes have been reported for separation, their catalytic functions and the coupled effects of metal incorporation on transport and reactivity under harsh conditions remain largely unexplored. A key challenge is the limited understanding of how metal incorporation simultaneously influences the membrane microstructure, gas transport, and catalytic function under high-temperature reaction conditions. We prepared Fe-embedded CMS membranes derived from 6FDA-DAM-DABA (3:2) polyimide through cocasting (Fe_cc), dip-coating (Fe_dp), and post-pyrolysis incipient wetness impregnation (Fe_IWI) to directly examine how the Fe incorporation pathway governs membrane microstructure, NH_3_ transport, and catalytic behavior. We probe the catalytic performance for NH_3_ synthesis and observed that all Fe/CMS membranes are active for NH_3_ production: Fe_cc/CMS exhibiting the highest steady-state turnover frequency (2.48 min^–1^), followed by Fe_dp (1.59 min^–1^) and Fe_IWI (1.23 min^–1^). Permeation, sorption, and diffusion measurements were employed to directly measure NH_3_ transport in CMS membranes with and without metals. Fe incorporation reduced NH_3_ permeability from 1484 Barrer (metal-free CMS) to 201–450 Barrer (Fe-containing CMS), demonstrating that Fe incorporation significantly alters NH_3_ transport through CMS structure under ambient permeation conditions. Additionally, electron microscopy, X-ray photoelectron spectroscopy, X-ray diffraction, Raman spectroscopy, and CO_2_ physisorption together revealed that the Fe incorporation pathway controls Fe particle size, oxidation state, and the extent of ultramicropore disruption within the CMS matrix. This work provides an integrated evaluation of gas transport and catalytic NH_3_ synthesis in Fe-CMS membranes and identifies the metal incorporation pathway as a critical design variable for catalytic membranes.

## Graphical abstract

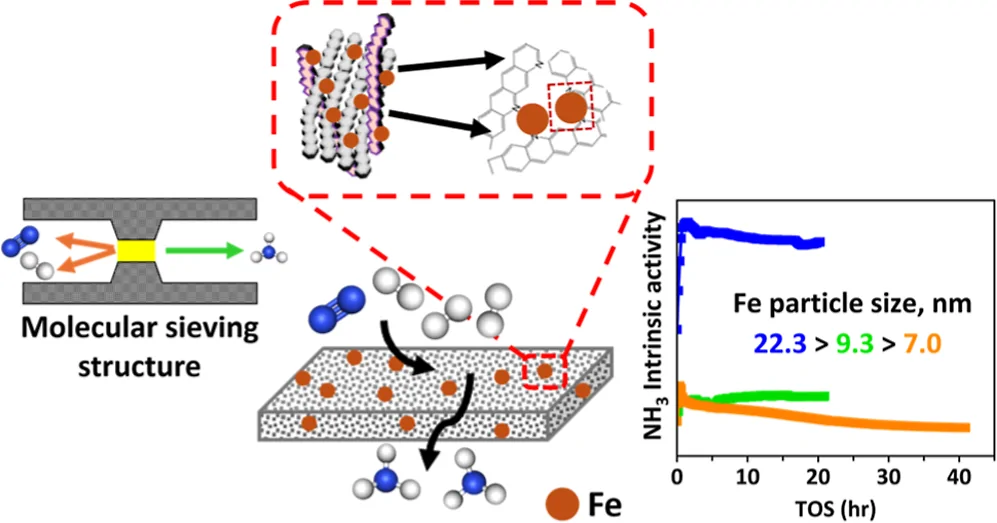

## 1 Introduction

Membrane reactors have been widely investigated in the chemical and petrochemical industries as a process-intensification strategy capable of integrating reaction and separation within a single unit. By selectively introducing reactants or removing products, membrane reactors can shift reaction equilibria, enhance conversion, and reduce downstream separation requirements.^1–3^ Prior demonstrations with Pt-impregnated alumina ceramic membranes and palladium membranes for alkane dehydrogenation highlighted the positive impact on product yield due to H_2_ removal by membrane reactors.^1,3^ Subsequent studies expanded this concept through flow-through catalytic membrane reactors, where sufficient reactant contact time and controlled permeation within catalytic pores led to significantly better performance.^4–6^ Building on these concepts, a diverse range of membrane materials have been explored in the literature, including microporous and sol–gel silica, metal–organic framework (MOFs), MXene-derived materials, polymers, zeolites, and carbon-based membranes.^7–16^ However, key challenges such as high temperature tolerance, mechanical durability, and long-term performance stability hinder large-scale development of catalytic membrane reactors.^17–19^

High-temperature operations (>500 °C) pose a specific challenge for the membrane reactor materials, since they tend to suffer from structural instability, sintering, or thermally induced stresses, resulting in performance loss.^19^ In addition, pressure gradients and thermal cycling can compromise mechanical integrity, while mismatches in thermal expansion between membrane and catalytic components complicate reactor integration.^17,18^ As a result, the development of membrane materials that combine thermal robustness, controlled transport, and catalytic functionality remains a central bottleneck for advancing catalytic membrane reactor technologies.

Here, carbon molecular sieve (CMS) membranes derived from polymer precursors represent a promising platform to address these challenges. CMS have shown excellent separation properties for a range of gas-pairs in prior literature.^20^ CMS membranes are synthesized from the pyrolysis of polymer membranes at temperatures >500 °C under inert gas. The resulting membranes exhibit a distinctive bimodal pore structure, comprising ultramicropores (*d* ≤ 0.7 nm) and micropores (0.7 nm < *d* < 2 nm).^21^ This unique structure enables the CMS membrane to simultaneously achieve high permeabilities and excellent molecular sieving selectivity among similar-sized gas molecules. Importantly, polyimide-derived CMS membranes have demonstrated excellent thermal stability under typical reaction (e.g., 500 °C for propane dehydrogenation) conditions.^22^ Additionally, CMS has emerged as an appealing platform for further functional modification, including the incorporation of metal species.^23–29^ Prior works employed metal-containing CMS membranes to utilize both molecular sieving and metal–gas interactions to enhance separation performance.^23–29^ Despite the demonstration of metal incorporation within CMS, the catalytic functionality of these materials has not been previously analyzed.

Embedding catalytically active metals within CMS membranes enables the development of dual-functional catalytic membranes, in which the CMS matrix governs gas transport, while the embedded metal provides catalytic activity. However, the incorporation of metals into CMS membranes introduces additional material complexities, which require detailed analyses. The high pyrolysis and reaction temperatures required for CMS formation can alter the oxidation state, dispersion, and morphology of the embedded metals. Furthermore, metal species may undergo restructuring during membrane synthesis or under catalytic reaction conditions, influencing both the activity and stability. Such structure–property relationships in metal-embedded CMS membranes remain poorly understood, and this work aims to bridge this gap by applying characterization tools relevant for traditional metal catalysts to these dual-functional materials.^30^

In this study, we use NH_3_ synthesis as a probe reaction to investigate the interplay between Fe incorporation strategy, membrane structure, transport behavior, and catalytic performance. NH_3_ synthesis remains industrially dominated by the Haber–Bosch process, which operates under harsh conditions (400–500 °C, 100–300 bar) to overcome equilibrium limitations.^31–33^ These conditions make NH_3_ synthesis particularly well suited for evaluating materials designed for high-temperature catalytic membrane reactors.

In this work, Fe-embedded CMS membranes were synthesized via three different metal incorporation strategies—cocasting, dip-coating, and post-pyrolysis incipient wetness impregnation. These strategies provide opportunities to study how metal introduction governs catalytic activity, gas transport, and membrane microstructure. By coupling NH_3_ synthesis reactivity measurements with detailed structural, chemical, and transport characterization, this work directly elucidates the interplay between Fe nanostructure, CMS pore accessibility, and diffusion limitations. This represents the first study explicitly designed to tailor and evaluate Fe-embedded CMS membranes for NH_3_ synthesis.

## 2 Materials and Methods

Detailed information regarding the materials and methods used in this work is provided in the Supporting Information file.

### 2.1 Membrane and Catalyst Synthesis

#### 2.1.1 Formation of Metal-Free Polymer Dense Film Membranes

The metal_free polymer dense films (Supporting Information, Table S1) were prepared from 6FDA-DAM-DABA (3:2) polyimide. The homogeneous solution of polymer precursor and tetrahydrofuran (THF) was cast in a N_2_-filled glovebag under a THF-rich atmosphere to ensure a uniform solvent evaporation rate. The polymer membranes were formed after having left the polymer solution for 3 days in the glovebag, followed by drying under vacuum at 120 °C for 24 h.

### 2.2 Fe-Embedded Carbon Molecular Sieve (Fe/CMS) Membranes

#### 2.2.1 Formation of Fe Cocasting (Fe_cc) Polymer Dense Film Membranes

Iron acetylacetonate (Fe­(acac)_2_) was dissolved in THF and mixed with specific wt % (Supporting Information, Table S1) of the polymer solution to prepare Fe cocast membranes. The mixed metal precursor and polymer solution were subsequently cocast following the same procedure as the metal_free polymer films.

#### 2.2.2 Formation of Fe Dip-Coating (Fe_dp) Polymer Dense Film Membranes

Iron acetylacetonate (Fe­(acac)_2_) (Supporting Information, Table S1) was dissolved in methanol and placed on a roller overnight to form a well-mixed dip-coating solution. The metal_free polymer membrane was immersed in this Fe_dp solution, and the container was covered with aluminum foil. This container was kept inside an inert N_2_-filled glovebag ensuring a methanol-rich atmosphere for 36 h. The polymer film was then rinsed with methanol and dried under vacuum at 120 °C for 24 h.

#### 2.2.3 Formation of CMS-Dense Film Membranes

The metal_free and Fe polymer membranes were pyrolyzed at 550 °C under Ar gas for 2 h (Figure 1e) to form the final CMS membranes.

**Figure 1:**
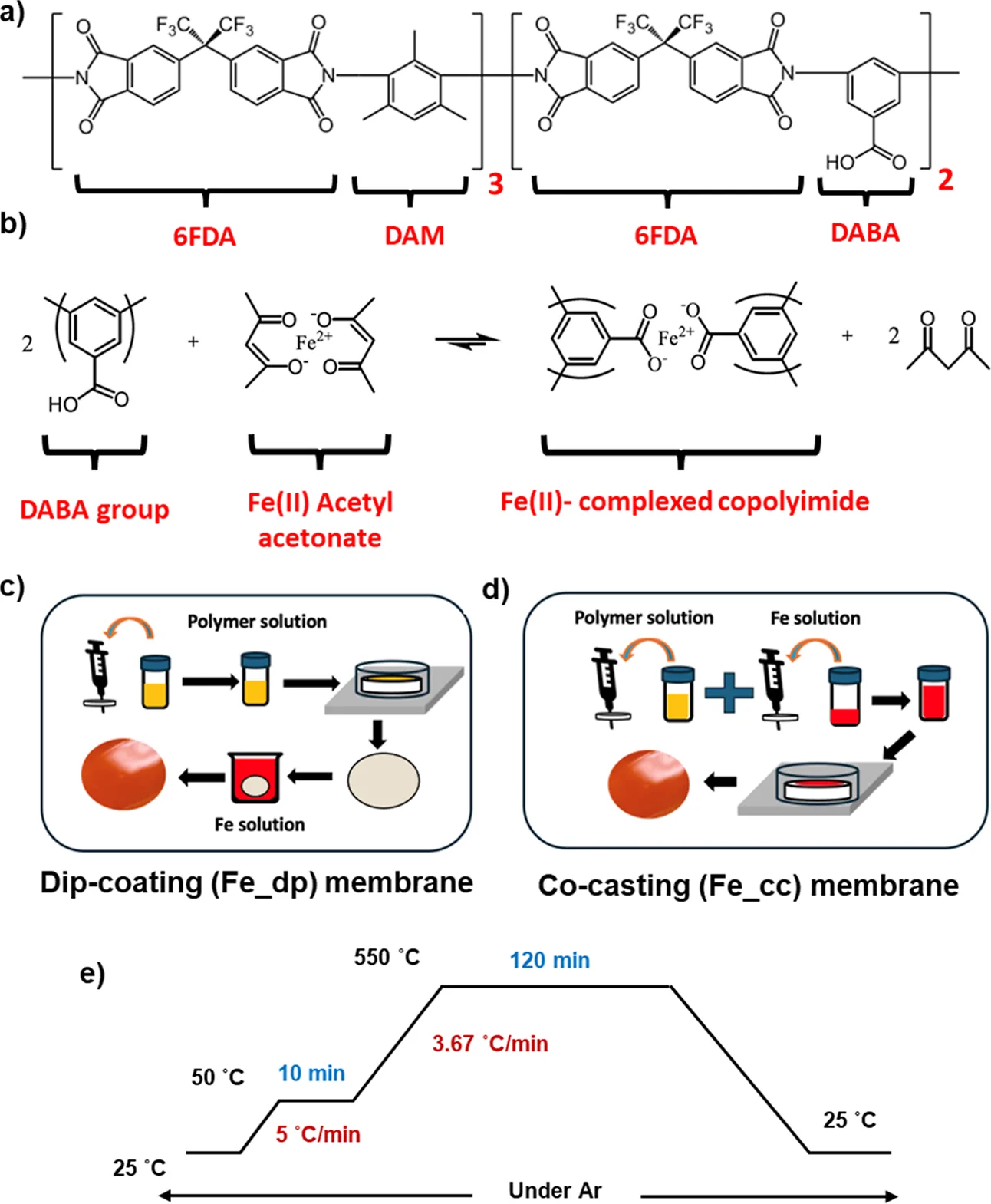
(a) 6FDA-DAM-DABA (3:2) polyimide structure, (b) envisioned cross-linking of Fe­(acac)_2_ with DABA groups in the polyimide, (c) dip-coating (Fe_dp) membrane casting method, (d) co-casting (Fe_cc) membrane casting method, and (e) pyrolysis process.

#### 2.2.4 Fe CMS Membranes via Incipient Wetness Impregnation (Fe_IWI/CMS)

A metal-free CMS membrane was crushed into a fine powder. Fe­(acac)_2_ was dissolved in methanol and then added dropwise to the powder metal-free CMS membrane. Fe_IWI/CMS was oven-dried at 120 °C for 30 min and then calcined under N_2_ at 500 °C for 4 h.

### 2.3 Conventional Fe Catalyst (Fe/C)

Activated carbon was treated in 4 M HNO_3_ and dried at 110 °C. Iron­(III) nitrate nonahydrate was used as a Fe precursor to synthesize the Fe catalyst supported on activated carbon using incipient wetness impregnation. The catalyst was dried at 110 °C and calcined under N_2_ at 500 °C for 4 h. The prepared 20 wt % iron catalyst is labeled “Fe/C”.

### 2.4 Performance of Fe/CMS Membranes

#### 2.4.1 Reactivity

Prior to the reactivity tests, membrane samples were stored in Petri dishes that were placed in a vacuum desiccator or glovebox. Ground membrane samples were diluted with silicon carbide at approximately 3 times the sample weight. The catalyst bed was supported between plugs of quartz wool in a stainless-steel tubular reactor. The desired temperature inside the reactor tube was controlled by a temperature controller connected to the tubular furnace via a K-type thermocouple ensuring its tip touched the top of the catalyst bed. The N_2_ and H_2_ gases were controlled using mass flow controllers capable of handling operating pressures up to 1500 psi. The samples were first pretreated at 480 °C for 6 h under stoichiometric mixture of H_2_/N_2_ (3:1) at a total flow rate of 30 mL/min. The ammonia synthesis reaction was carried out at 500 °C and 400 psi, keeping the same flow rates used for the pretreatment step. The high pressure inside the reactor was maintained using a downstream back pressure regulator. The reaction gas mixture was quantified using a Micro GC gas chromatography system. Typically, the experiment was run for a minimum of 20 h time on stream (TOS) or until steady state was achieved.

#### 2.4.2 Sorption Measurements

Equilibrium sorption isotherms for CMS membranes were measured using a pressure decay sorption setup operated at 35 °C (Figure S1). CMS samples were degassed under vacuum and exposed to NH_3_, with a pressure transducer monitored over time to quantify gas uptake based on the system mole balance. Longer equilibration times were required for NH_3_ for each pressure point due to its condensable nature. The resulting sorption data were analyzed using a dual-mode sorption model to extract Henry, Langmuir, and affinity parameters for each gas. The gas sorbed by the membrane was calculated based on mole balance equation ref, and the sorption coefficients are defined as the penetrant concentration of A (*C*_A_) sorbed by the materials at the equilibrium fugacity (*f*_A_).

$$S_{A}=\frac{C_{A}}{p_{A}}=k_{C,A}\times f_{A}+\frac{C_{L,A}^{\prime }\times b_{A}}{1+b_{A}\times f_{A}}$$

where *C*_L,A_^′^ is the Langmuir coefficient, *b_A_* is the Langmuir affinity, and *k*_C,A_ is the continuous mode’s sorption constant at equilibrium fugacity (*f*_A_), where *f*_*A*_ = partial pressure (*p*_*A*_) × fugacity coefficient (Φ_*A*_). As NH_3_ is the highly condensable gas at testing conditions, Φ_*A*_ varies with pressure. Φ_*A*_ was calculated for each gas using CHEMCAD.

#### 2.4.3 Permeation Measurements

Pure gas permeation measurements for CMS dense film membranes were conducted using a constant volume permeation system at 35 °C (Figure S3). Permeation experiments were performed under an upstream pressure of 30 psi and downstream under vacuum (<1 Torr) following a specific stepwise protocol to reduce the chance of breaking the CMS membranes. Gas permeability was calculated from the downstream pressure rise, and the membrane thickness and area were measured by a digital microscope. The results are reported in Barrer.

1 $$P=\frac{1.764\times 10^{6}\times V\times l}{\left(T+273.15\right)\times A\times \Delta p}\times \left(\frac{dp}{dt}\right)$$

where *P* is the permeability (Barrer), *V* is the downstream volume (cm^3^), *l* is the membrane thickness (mil), $\left(\frac{dp}{dt}\right)$ is the downstream pressure rising rate (Torr/s), *A* is the area of the membrane for permeation (cm^2^), and Δ*p* is the partial pressure difference across the membrane (psi).

The membrane area and membrane thickness were measured by a digital microscope (Dunwell Tech, Inc., Torrance, CA, no. AF7515MZT).

#### 2.4.4 Transport in CMS Membranes

Permeability and selectivity are the two key factors commonly used to characterize CMS membranes. Additionally, CMS membranes follow a sorption–diffusion model like other molecular sieve membranes wherein gas molecules first sorb on the upstream side of the membrane and then are transported to the downstream side of the membranes by the chemical driving forces and desorb. The permeability (*P*_A_) of component A is defined by eq 2:

2 $$P_{A}=\frac{N_{A}\times l}{\Delta p_{A}}$$

where *N*_A_ is the constant flux of penetrant A through membranes thickness *l* by the partial pressure difference of penetrant A (Δ*p*_A_). Permeability is also described as an average product of diffusion coefficient (*D*_A_) and thermodynamic sorption coefficient (*S*_A_), determined by eq 3:

3 $$P_{A}=D_{A}\times S_{A}$$

The diffusion coefficient (*D*_A_) is directly calculated from permeability (*P*_A_) divided by sorption (*S*_A_), eq 4:

4 $$D_{A}=\frac{P_{A}}{S_{A}}$$

### 2.5 Materials Characterization

#### 2.5.1 Inductively Coupled Plasma Optical Emission Spectrometry (ICP-OES)

The actual metal loading of all membrane and catalyst samples was quantified by an Agilent 5800 ICP-OES. In all samples, Fe was extracted using either aqua regia or microwave acid digestion.^34^

#### 2.5.2 X-ray Diffraction (XRD)

XRD patterns of the CMS membranes were obtained on a PANalytical X’Pert Pro X-ray Diffractometer.

#### 2.5.3 X-ray Photoelectron Spectroscopy

The X-ray photoelectron spectra of all samples were collected using a Physical Electronics PHI 5000 VersaProbe X-ray Photoelectron Spectroscope and a Kratos Axis Supra+ X-ray Photoelectron Spectrometer.

#### 2.5.4 CO_2_ Physisorption

The porosity measurements on all samples were performed using CO_2_ physisorption at 0 °C on a Micromeritics ASAP 2060+.

#### 2.5.5 Scanning Transmission Electron Microscopy

The high-angle annular dark-field scanning transmission electron microscopy (HAADF-STEM) imaging of Fe_cc/CMS and Fe_dp/CMS was conducted on a cross-section sample using a Nion UltraSTEM100, and the TEM images for Fe_IWI/CMS and Fe/C were obtained using a JEOL 2200FS.

#### 2.5.6 Raman Spectroscopy

Raman spectroscopy was performed on the CMS membrane samples using a Renishaw inVia Raman microscope.

#### 2.5.7 H_2_ Chemisorption

The Fe site density measurements on all samples were performed using H_2_ chemisorption at 100 °C on a Micromeritics ASAP 2020+.

## 3 Results and Discussion

Fe was incorporated into CMS membranes using three methods, and the resulting Fe_cc/CMS, Fe_dp/CMS, and Fe_IWI/CMS materials were evaluated against a conventional Fe/C catalyst for NH_3_ synthesis and transport. The Fe on the activated carbon (Fe/C) catalyst was prepared with 20% Fe loading using incipient wetness impregnation and used as a control sample. The actual Fe loadings in the CMS membranes and the Fe/C catalyst were quantified using ICP-OES (Table 1).

**Table 1:** Fe Loading in CMS Membranes and Activated Carbon Support

samples	Fe loading (ICP-OES), wt %
Fe_dp/CMS membrane	1.0
Fe_cc/CMS membrane	2.0
Fe_IWI/CMS	1.2
Fe/C	19.2

### 3.1 Effects of Fe Incorporation on Reaction and Separation Performance of CMS Membranes

#### 3.1.1 Catalytic Performance of Fe/CMS Membranes and Fe/C

The NH_3_ production rates for Fe-CMS (Fe_cc/CMS, Fe_dp/CMS, and Fe_IWI/CMS) membranes, the metal-free CMS membrane, and a traditional Fe/C catalyst are shown in Figure 2. All Fe-containing materials exhibit measurable NH_3_ production activity over the entire TOS. The steady-state NH_3_ formation rates span a broad range, from ∼250 to 1350 μmol/(g_cat_ h), with varied trends in activity over time.

**Figure 2:**
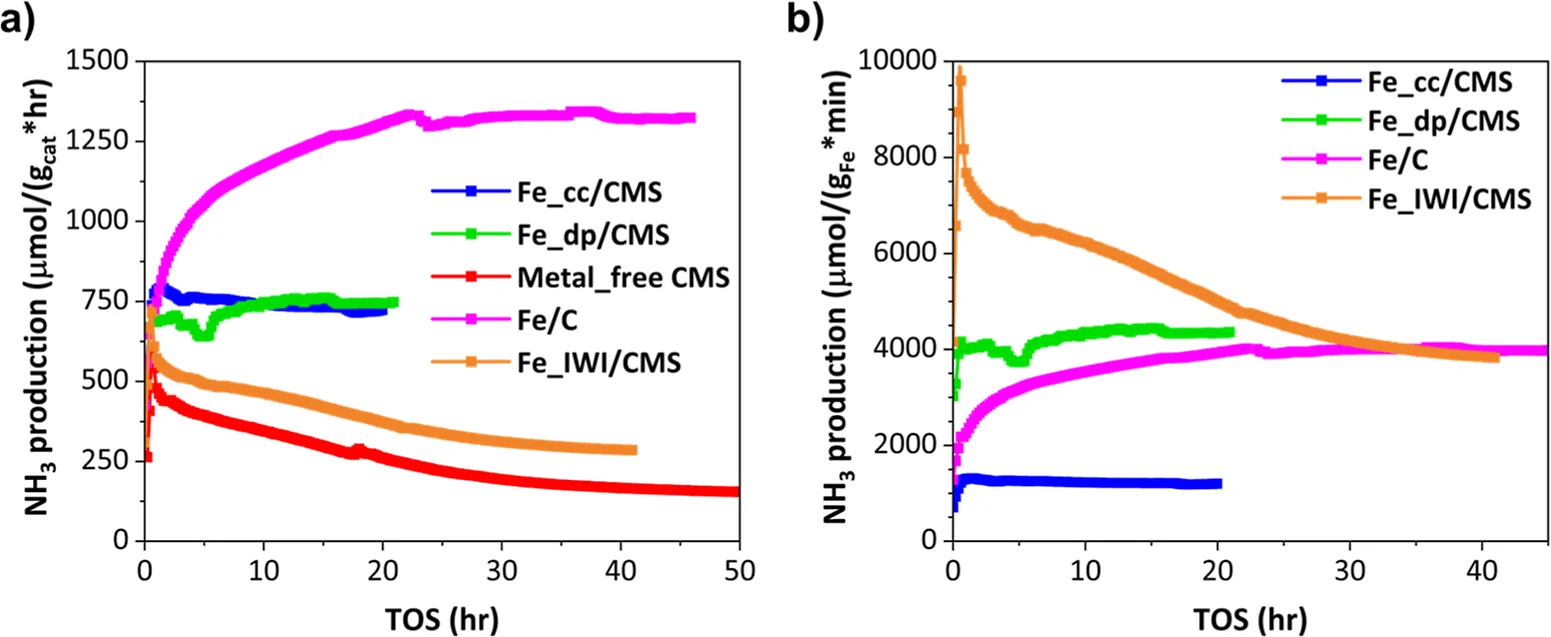
NH_3_ production rate (a) normalized by catalyst mass and (b) normalized by metal content for Fe/CMS membranes, Fe/C catalyst, and metal-free CMS. Reaction conditions: 500 °C, 400 psi, total flow: 30 mL/min (3:1 H_2_/N_2_). TOS = time on stream.

The Fe/C catalyst shows a continuous increase in NH_3_ production rate, going from ∼400 to 1250–1350 μmol/(g_cat_ h) after ∼20 h TOS. In contrast, the Fe_dp/CMS and Fe_cc/CMS membranes exhibit a relatively stable NH_3_ production rate of ∼750 μmol/(g_cat_ h). Fe_IWI/CMS displays a gradual decrease in NH_3_ production rate, decreasing from ∼700 to <300 μmol/(g_cat_ h) over 40 h TOS. The metal-free CMS shows lower activity, as expected, but follows a similar deactivation trend, with rates decreasing from ∼585 to <200 μmol/(g_cat_ h) over time. The steady-state values for all materials are reported in Table S4.

When normalized by the Fe content (Figure 2b) measured with ICP-OES (Table 1), Fe_dp/CMS exhibited the highest steady-state NH_3_ production of ∼4400 μmol/(g_Fe_ min), while the Fe/C exhibited 4000 μmol/(g_Fe_ min). Fe_cc/CMS exhibited the lowest NH_3_ production of 1200 μmol/(g_Fe_ min). The maximum activity observed for Fe_IWI/CMS was ∼9900 μmol/(g_Fe_ min) in the initial hour, but it later decreased to <4000 μmol/(g_Fe_ min) after 30 h on stream.

These observed differences and trends in the activities of various Fe/CMS samples suggest that the different synthesis methods for Fe_cc/CMS, Fe_dp/CMS, and Fe_IWI/CMS have an impact on Fe structures, which directly affect the reaction performance. We also note that the high activity of Fe/C and the low activity of the metal-free CMS indicate that the activity observed over Fe_dp/CMS and Fe_cc/CMS is predominantly associated with Fe and not the CMS. The CMS has a small but nonzero contribution to the overall catalytic performance. The observed NH_3_ production rate per gram of catalyst for Fe_IWI/CMS was slightly higher than the metal-free CMS but lower than Fe/C. This is expected since Fe_IWI/CMS only had 1.2 wt % Fe loading, while the Fe/C catalyst had ∼20% loading. The deactivation trend of Fe_IWI/CMS over time mirrors that of the metal-free CMS sample, which shows a similar decline in NH_3_ production rate. This suggests that the Fe_IWI/CMS sample retains the bulk properties of the underlying CMS, with some additional activity arising from the impregnated Fe on the surface. Interestingly, while the performance trends for Fe/C and Fe_IWI/CMS materials differ significantly, they appear to converge to ∼4000 μmol/(g_Fe_ min) after >30 h. In comparison to the Fe_IWI/CMS membranes, the Fe_cc/CMS and Fe_dp/CMS samples showed stable NH_3_ production rates over time. These observed variations in activity with the different synthesis methods highlight the important role of the underlying support and the metal incorporation strategy. This is further investigated in the subsequent sections of this paper, wherein various characterization tools such as X-ray photoelectron spectroscopy (XPS), high-resolution TEM (HR-TEM), and chemisorption are used to probe the metal structure (effective size and dispersion) and oxidation states. Given the stable NH_3_ production observed for Fe_cc/CMS and Fe_dp/CMS, the NH_3_ permeability was examined to further probe the role of Fe in the overall structure–function relationships. These transport-based studies, along with the results from the characterization techniques, provide insights into the catalytic performance and stability of these materials.

#### 3.1.2 Ambient Permeation and Sorption Measurements to Probe Fe-CMS Membrane Structures

Pressure-decay sorption measurements under ambient temperatures (35 °C) provide insights into how Fe incorporation leads to redistribution of accessible free volume between the two transport domains—Henry and Langmuir in CMS. CMS membranes are envisioned as networks of aromatized polymer strands that organize into plate-like structures, which further assemble into an amorphous morphology. The voids between adjacent plates are microporous and serve as the primary sorption sites for gas molecules, while the interstrand slit-like regions form ultramicropores that govern the molecular-sieving behavior and selectivity of the membrane. Sorption within the microporous voids is commonly described by a Langmuir mechanism. In addition to these Langmuir-type domains (L), CMS membranes are also envisioned to contain a continuous domain composed of irregularly packed aromatic strands separated by ultramicroporous voids. Sorption in this continuous domain (C) follows Henry’s law behavior, exhibiting a linear dependence on gas partial pressure.

The NH_3_ sorption isotherms for the metal free CMS, Fe_cc/CMS, and Fe_dp/CMS at 35 °C are presented in Figure 3. These isotherms display a characteristic dual-mode sorption behavior, in which the total uptake arises from the combined contribution of the C-domain and the L-domain.^35,36^ To understand the effects of Fe on sorption performance in CMS membranes, the experimental sorption isotherms were fit to the dual mode sorption model, and the parameters were estimated using “fminsearch” function in MATLAB with the constraints *k*_C,A_, *C*_L,A_^′^, and *b* > 0 (Table 2). The affinity constant “*b*” remains essentially unchanged in the metal containing samples and the metal free samples, showing that Fe does not substantially affect the NH_3_–surface interaction energy. The incorporation of Fe leads to a ∼10% reduction in total sorption capacity (*C*_L,A_^′^) in the Fe_cc/CMS and Fe_dp/CMS samples, in comparison to the metal-free CMS samples, which is observed in the NH_3_ sorption isotherms. Furthermore, the retained Langmuir-type behavior after Fe incorporation is beneficial because it indicates that the overall CMS structure and associated microporous sorption sites responsible for gas selectivity are largely preserved after Fe incorporation. This suggests that Fe primarily affects the ultramicroporous regions of the CMS samples which are not reflected in these sorption measurements. The diffusion coefficient calculations and Brunauer–Emmett–Teller (BET) isotherm results shown in the later sections support this hypothesis.

**Figure 3:**
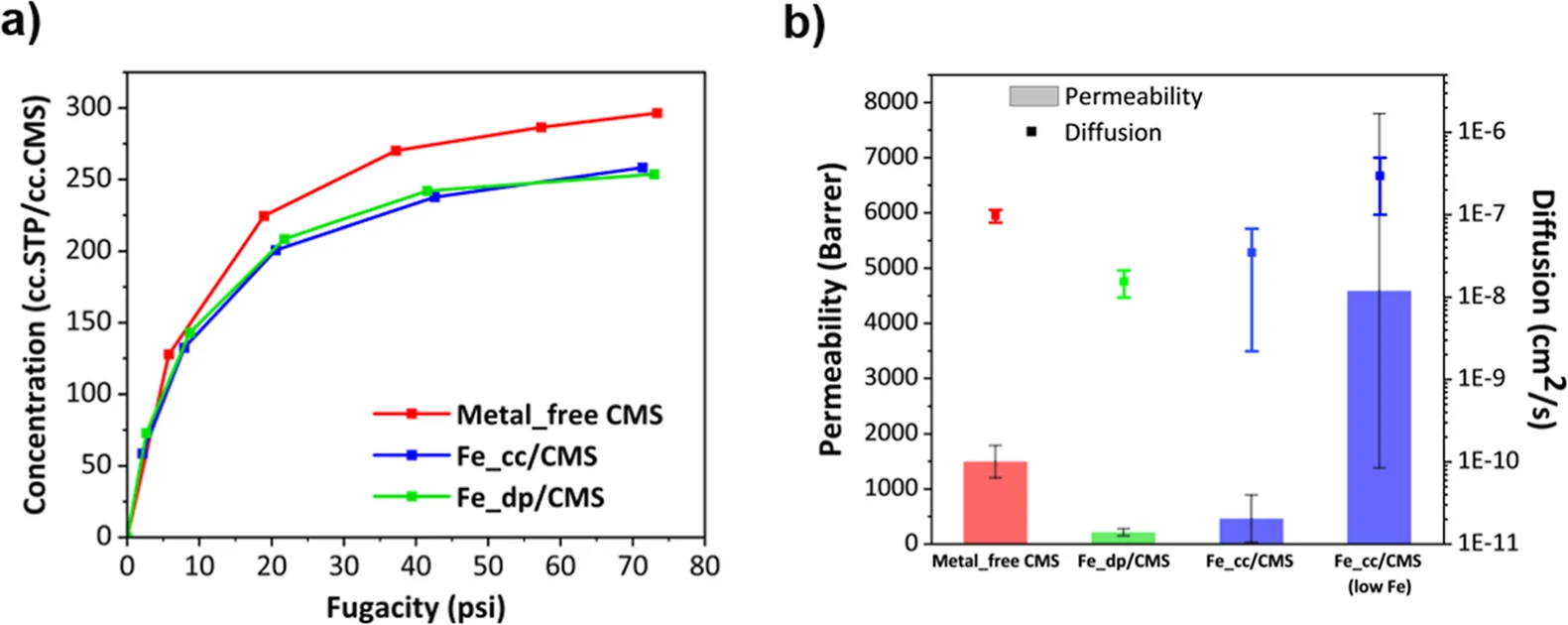
NH_3_ sorption isotherms for (a) metal_free CMS (blue), Fe_cc/CMS (red), Fe_dp/CMS (green) membranes at 35 °C, and (b) overall NH_3_ transport through CMS membranes used in this study.

**Table 2:** Dual Mode Sorption Parameters for the NH_3_ Sorption Isotherm at 35 °C in Different CMS Samples

	*k*_C,A_ (cc STP/cc CMS psi)			*C*_L,A_^′^ (cc STP/cc CMS)			*b*_A_(1/psi)		
gas	metal_free	Fe_cc/CMS	Fe_dp/CMS	metal_free	Fe_cc/CMS	Fe_dp/CMS	metal_free	Fe_cc/CMS	Fe_dp/CMS
NH_3_	0.01	0.02	0.02	317	287	285	0.11	0.11	0.12

NH_3_ permeation through CMS membranes under similar ambient conditions (35 °C) was evaluated to elucidate how Fe incorporation and the resulting structural changes influence the gas transport behavior. In this section, the NH_3_ permeabilities in different CMS membranes (with and without metal) are tabulated in Table 3. During the preliminary tests, it was noted that CMS prepared with a 3 wt % polymer solution faces an embrittlement problem, leading to failure at feed pressures above 10 psi. To ensure mechanical integrity for permeation measurements at ≥30 psi, CMS membranes were prepared using a 5–7 wt % polymer solution, while 3 wt % solution was used for the reaction and sorption experiments and all material characterizations. However, attempts to proportionally increase the Fe loading in Fe_cc/CMS from 5 to 7 wt % polymer solution resulted in dope gelation, as well as casting defects such as curling, bubbles, and pinholes (Figure S4), which compromised the membrane integrity. Ideally, to maintain the same polymer/metal ratio as in the case of Fe_cc/CMS from 3 wt % polymer, ∼0.14 g of Fe precursor in 5 mL of THF should be used. However, to avoid defect formation and ensure reliable casting, the amount of Fe in the precursor solutions was lowered. Two samples with different Fe loadings were prepared, namely, Fe_cc/CMS (0.07 g of Fe precursor) and Fe_cc/CMS_low-Fe (0.03 g of Fe precursor), as listed in Table 3. As shown in the table, this caused the theoretical polymer/metal ratio to be different in the permeation samples as compared to sorption and characterization samples. Even though the permeation samples cannot be directly correlated with the sorption and characterization samples because of these differences in metal loading, the relative trends among the samples and Fe incorporation methods remain informative. Future work will therefore focus on a hollow fiber format, which can better withstand high pressure and enable more consistent comparisons of permeation, sorption, and characterization structural properties across samples.

**Table 3:** Casting Solution Formulation for Fe_dp/CMS and Fe_cc/CMS Permeation Samples^a^

samples	polymer solution	metal solution	polymer/Fe precursor ratio	Fe loading (ICP-OES), wt %
Fe_dp/CMS	20 mL THF + ∼1 g polymer precursor	40 mL THF + 3.7 g Fe(acac)_2_	1:3.70	0.44
Fe_cc/CMS	20 mL THF + ∼1 g polymer precursor	5 mL THF + 0.07 g Fe(acac)_2_	1:0.07	1.1
Fe_cc/CMS_low-Fe	20 mL THF + ∼1 g polymer precursor	5 mL THF + 0.03 g Fe(acac)_2_	1:0.03	-
Comparison with Sorption and Characterization Samples				
Fe_dp/CMS	20 mL THF + ∼0.5 g polymer precursor	40 mL THF + 3.7 g Fe(acac)_2_	1:7.4	1.0
Fe_cc/CMS	20 mL THF + ∼0.5 g polymer precursor	5 mL THF + 0.07 g Fe(acac)_2_	1:0.14	2.0

a All CMS membrane compositions were synthesized using the same 550 °C pyrolysis protocol.

Gas permeability in CMS membranes reflects the coupled effects of sorption and diffusion. The measured NH_3_ permeability at 35 °C and 30 psi for the set of CMS membranes reveals a clear dependence on the Fe incorporation strategy (Figure 3b). For the metal_free CMS, the high NH_3_ permeability suggests that diffusion occurs through the well-connected ultramicroporous network that remains accessible for NH_3_. In contrast, both Fe_dp/CMS and Fe_cc/CMS membranes exhibit substantially reduced NH_3_ permeabilities, demonstrating a disruption in the effective diffusion pathway rather than sorption effects alone. The most pronounced reduction is observed for Fe_dp/CMS, which exhibits the lowest NH_3_ permeability (201 Barrer) in comparison to Fe_cc/CMS (450 Barrer) and metal_free (1484 Barrer). The incorporated Fe nanoparticles are envisioned to lead to constricted pathways which significantly reduces NH_3_ diffusion. In case of Fe_dp/CMS, a 10× reduction in the diffusion coefficient is observed in comparison to the metal_free CMS, indicating that such constricted pathways are present in a higher concentration in Fe_dp/CMS vs Fe_cc CMS. This observation is also reinforced by the CO_2_ physisorption data (Figure 8a), wherein the Fe_dp/CMS membranes showed the lowest ultramicropore (<0.7 nm) distributions among all the CMS membranes.

On the contrary, Fe_cc/CMS membranes display intermediate NH_3_ permeation but exhibit greater variability across replicate samples. This behavior suggests heterogeneity in the Fe nanoparticle distribution, which is also consistent with the broad particle size distribution observed for these samples with HR-TEM (discussed in the context of Table 5 and Figure 6 below). Attempts to mitigate these effects by reducing the Fe loading (0.03 g) (Fe_cc/CMS_low-Fe) did not result in elimination of the variability in NH_3_ permeation. It is therefore evident that NH_3_ transport in CMS membranes is highly sensitive to subnanometer pore constriction. It should be noted that the ambient permeation and sorption measurements in this study are intended primarily to be used as tools to probe the impacts of incorporated Fe on the resulting CMS structures. For practical use as membrane reactors, detailed analysis of the CMS membrane separation properties, i.e., NH_3_ permeability and NH_3_/H_2_ and NH_3_/N_2_ selectivities, must be measured under NH_3_ synthesis temperature and pressure conditions.

#### 3.1.3 Stability of CMS Membranes before and after NH_3_ Synthesis

To assess the stability of the materials, pressure decay sorption measurements were collected on the fresh and post-reaction samples. As part of this, the Fe_cc CMS samples were exposed to reaction conditions (i.e., 3:1 H_2_/N_2_ (24:8 sccm) at *T* = 500 °C and pressure 15 bar) for ∼8 h, and the sorption isotherms and the fitted parameters were compared against the fresh Fe_cc CMS samples. As shown in Figure 4a and Table 4 compared to the Fe_cc/CMS fresh sample, the Fe_cc/CMS post-reaction samples displayed a 1.7× reduction in *C*_L,A_^′^, while *k*_C,A_ increased 85× for NH_3_. This clearly shows the impact of reaction conditions on the redistribution of the Langmuir and Henry domains. To determine whether these changes arise from the presence of Fe or from the reaction environment itself, a parallel study was performed on metal-free CMS exposed to the same NH_3_ synthesis reaction conditions. As shown in Figure 4b and Table 4, the metal-free CMS exhibited similar sorption behavior, suggesting that Fe is not the driving factor for this change and the CMS structure itself shows some structural rearrangement in the presence of the reaction mixture.

**Figure 4:**
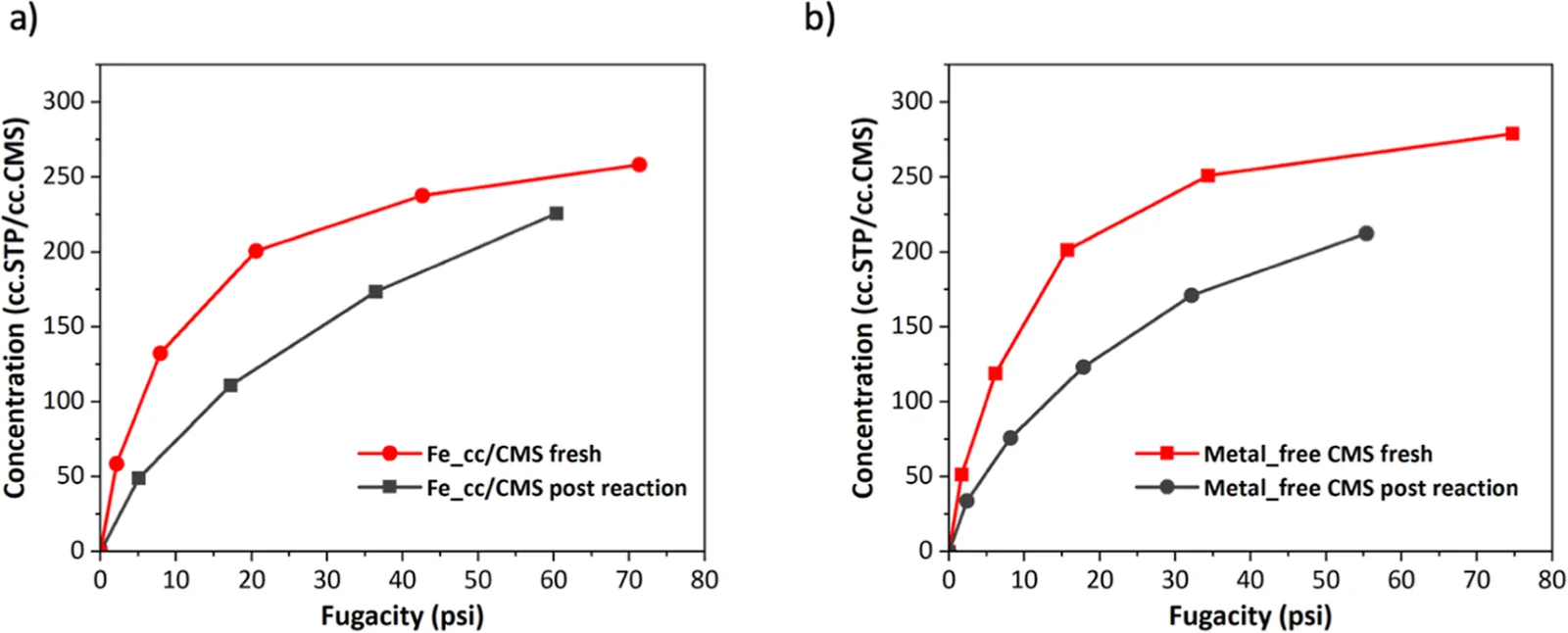
NH_3_ sorption isotherms at 35 °C for (a) metal_free CMS fresh and post-reaction samples and (b) Fe_cc/CMS fresh and post-reaction samples.

**Table 4:** Dual Mode Sorption Parameters for NH_3_ at 35 °C for Fe_cc/CMS Fresh and Post-Reaction Samples and Metal_Free CMS Fresh and Post-Reaction Samples Derived from 6FDA-DAM-DABA (3:2) Polyimide by Pyrolysis at 550 °C Ultra-High Purity Ar

	*k*_C,A_ (cc STP/cc CMS psi)		*C*_L,A_^′^ (cc STP/cc CMS)		*b* (1/psi)	
sample	fresh	post-reaction	fresh	post-reaction	fresh	post-reaction
Fe_cc/CMS	0.02	1.70	287	162	0.11	0.07
metal_free CMS	0.01	1.50	317	160	0.11	0.09

To further investigate the reasons that caused the structural rearrangement, XPS analysis was performed to determine the sp^3^/sp^2^ ratio for fresh Fe CMS and post-reaction Fe CMS. The results showed the rearrangement of sp^3^ and sp^2^ configurations for post-reaction Fe_IWI/CMS, Fe_dp/CMS, and Fe_cc/CMS samples compared to fresh samples (Figure S12 and Table S7). This rearrangement of sp^3^ and sp^2^ carbon is envisioned to disrupt the plate-like structure (micropores) and could lead to an internal rearrangement between the two domains, L and C. This scenario would naturally lead to a decline in *C*_L,A_^′^ and increase in *k*_C,*A*_ as observed from the NH_3_ sorption isotherms. Ma et al. demonstrated that sp^3^/sp^2^ ratio in the CMS membrane could change under a H_2_ content (4% of H_2_ in H_2_/Ar mixture) above 500 °C.^37^ They attributed this structural change to interactions between H_2_ and unsaturated carbon structures, which promoted the formation of additional carbon branching and altered the resulting pore structure. It is therefore possible that similar changes occur in our materials when exposed to the reaction mixture of 75% H_2_/25% N_2_ at 500 °C. To complement the sorption and XPS results, and test this proposed hypothesis, we conducted BET CO_2_ physisorption for both the fresh Fe_cc/CMS and the H_2_-exposed Fe_cc/CMS (Figure S8) samples, and the results are consistent with sorption isotherm and XPS results, showing a decrease in pore volume after H_2_ exposure. This clearly demonstrates that H_2_ exposure (Figure 5) is the leading cause of decline in the Langmuir domain capacity of the membranes and the overall structural changes associated with the reaction environment.

**Figure 5:**
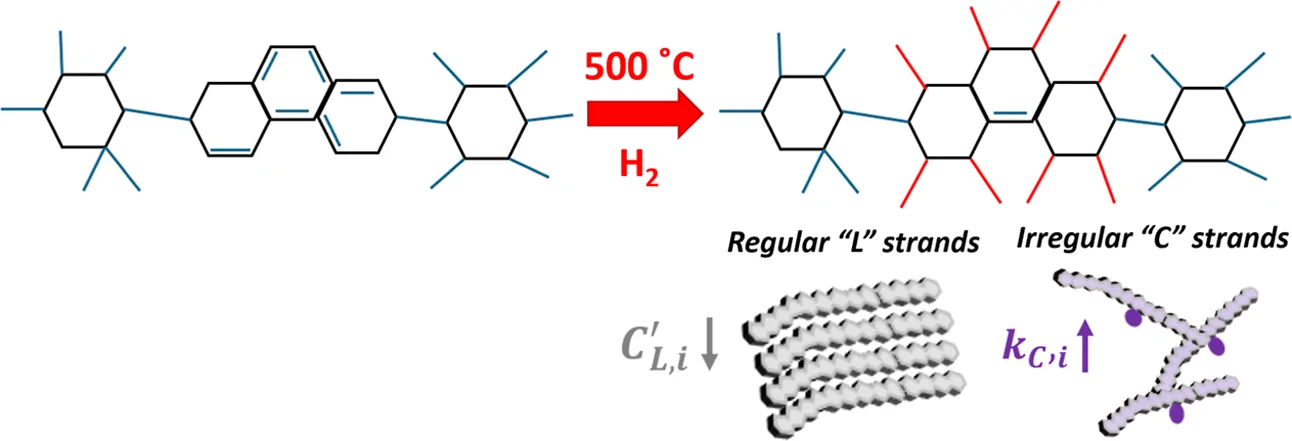
Possible mechanism of the H_2_ environment impact on the CMS membrane structure at 550 °C.

### 3.2 Metal Structure and Chemical State of Fe within CMS Membranes

As the average size, distribution, and dispersion of the Fe nanoparticles within the CMS membranes play a key role in the reaction performance, TEM images were obtained to compare these properties across the different metal samples. The representative images for (a) Fe_cc/CMS, (b) Fe_dp/CMS, (c) Fe_IWI/CMS, and (d) Fe/C are shown in Figure 6. The cross-sectional view of the metal-free membrane (Figure S12) confirms the absence of Fe particles. The particle size analysis showed that the Fe nanoparticles in Fe_dp/CMS have an average size of 9.3 ± 5.3 nm, while Fe_cc/CMS had an average particle size of 22.3 ± 11.4 nm (Figure S11). These average particle sizes correspond to a dispersion of ∼12 and ∼5% for Fe_dp/CMS and Fe_cc/CMS, respectively (Table 5). The average particle size and dispersion for Fe_IWI/CMS, on the other hand, were measured to be 7.0 ± 2.6 nm and 15.8%, which is similar to that of the Fe_dp/CMS membrane. In fact, Fe_IWI/CMS and Fe_dp/CMS both exhibit similar steady-state NH_3_ production values, although the former shows significant deactivation during TOS measurements.

**Figure 6:**
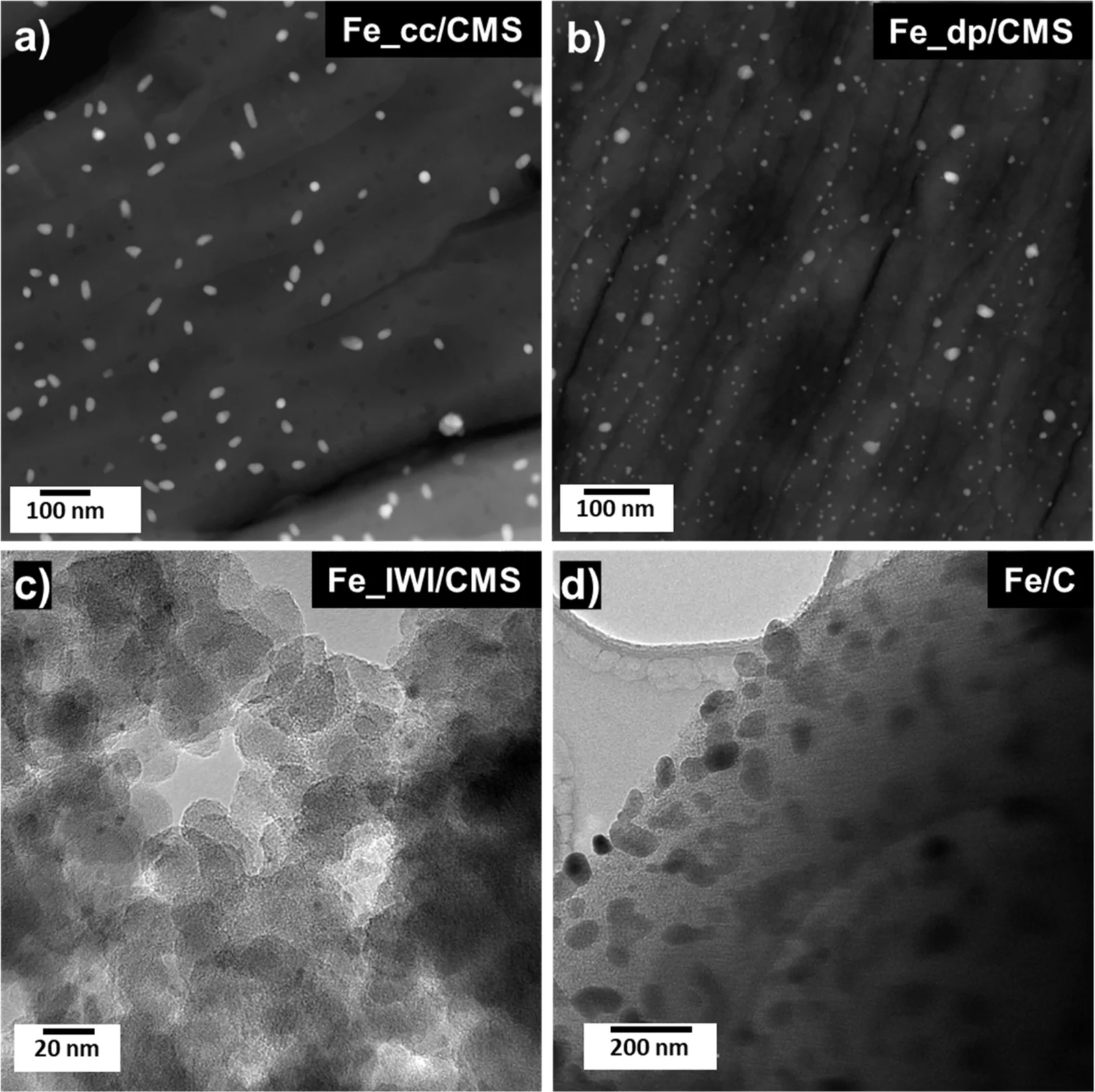
HAADF-STEM cross-sectional surface images for (a) Fe_cc/CMS, (b) Fe_dp/CMS, and bright-field TEM images for (c) Fe_IWI/CMS and (d) Fe/C.

**Table 5:** S/TEM Particle Size Analysis and H_2_ Chemisorption Data for Samples Used in This Study

	STEM/TEM		H_2_ chemisorption	
samples	average particle size, nm (#)^a^	metal dispersion, %	site density, μmol/g	TOF, min^–1^ ^b^
Fe_dp/CMS membrane	9.3 ± 5.3 (543)	11.8	7.85 ± 2.19	1.59
Fe_cc/CMS membrane	22.3 ± 11.4 (254)	4.9	4.90 ± 4.56	2.48
Fe_IWI/CMS membrane	7.0 ± 2.6 (79)	15.8	3.90 ± 1.70	1.23
Fe/C catalyst	28.6 ± 12.7 (133)	3.8	–	–

a Particles analyzed.

b Turnover frequency (TOF) at steady state calculated using site densities obtained from H_2_ chemisorption.

The smaller particle size and higher dispersion observed for the Fe_dp/CMS material suggest that the dip-coating synthesis route limits extensive Fe aggregation. When these materials are synthesized by dip coating (Fe_dp/CMS), the intrinsic transport resistance of the polymer membrane limits the penetration of larger Fe species, leading to the formation of smaller Fe nanoparticles. Furthermore, the cocasting route promotes direct interaction between Fe species and the available DABA groups during solution mixing. Since the number of DABA groups in the polymer film is finite, Fe can exceed the available anchoring capacity of DABA groups, leading to poorly stabilized Fe species that cluster during pyrolysis. This interpretation is consistent with the larger particles and lower dispersion observed for the Fe_cc/CMS material.

The large difference in particle size across the set of materials rationalizes the differences in the NH_3_ synthesis rates. The TOF, or active site normalized rate, of the Fe/CMS samples was calculated by normalizing the NH_3_ production rates using Fe active site density from H_2_ chemisorption (Table 5). It should be noted that the H_2_-chemisorption-derived site densities represent H_2_-accessible Fe sites under the specific pretreatment conditions and may not directly correspond to the Fe sites present under NH_3_ synthesis conditions. The highest TOF was obtained over Fe_cc/CMS (2.48 min^–1^), followed by Fe_dp/CMS (1.59 min^–1^) and Fe_IWI/CMS (1.23 min^–1^). This observed intrinsic behavior of Fe particles is consistent with the previously reported structural sensitivity of Fe-catalyzed NH_3_ synthesis; the material with the largest Fe particles exhibits the highest TOF.^38,39^

The broad particle size distribution in Fe_cc/CMS directly contributes to sample-to-sample variability, resulting in large standard deviations in permeability measurements, as discussed earlier. The porosity of the underlying support and the method used to incorporate Fe also play a significant role in determining the properties. The catalytic stability also depends on the Fe incorporation strategy. As shown in Figure 2, Fe_cc/CMS and Fe_dp/CMS exhibit comparatively stable NH_3_ production over the evaluated TOS, whereas Fe_IWI/CMS shows a substantially greater decline in activity. The instability observed for the Fe_IWI samples over TOS under reaction conditions may be attributed to the limited availability of DABA moieties remaining after pyrolysis to anchor Fe. This likely leads to poorly anchored Fe species, affecting the particle stability and reducing the number of active sites. In contrast, although Fe/C exhibits the highest metal loading compared with Fe_cc and Fe_dp/CMS, its NH_3_ production per gram of Fe is lower than that of Fe_dp/CMS. This suggests that the CMS membrane structure promotes more effective gas transport and better utilization of Fe active sites, leading to a higher NH_3_ production per unit mass of Fe.

The Fe-containing CMS membranes, Fe_cc, Fe_dp, and Fe_IWI, were analyzed by XPS to understand the initial oxidation states of Fe as shown in Figure 7. The deconvoluted Fe 2p spectra for Fe_cc/CMS (Figure 7 a) showed peaks at 707.0 and 719.9 eV resembling Fe^0^, while peaks at 709.7 and 722.8 eV were consistent with Fe^2+^.^40^ For Fe_dp/CMS Figure 7b, peaks observed at 707.8 and 721.4 eV were assigned to Fe^0^, while peaks at 709.3 and 722.9 eV were assigned to Fe^2+^. Additional peaks observed at 711.6 and 726.0 eV were attributed to Fe^3+^. The deconvoluted spectra for both Fe_IWI/CMS and Fe/C showed peaks at 711.3 and 724.4 eV and also attributed to Fe^3+^.^34^ These observed differences in the oxidation states of Fe between materials suggest that the cocasting and dip-coating methods influence the bond formation between Fe complexes and the DABA group, thus affecting the oxidation state of Fe. Fe_cc/CMS contains a more reduced phase distribution, mainly as Fe^0^ and Fe^2+^, with no significant Fe^3+^ contribution after pyrolyzing under Ar at 550 °C, which may provide more active sites for NH_3_ synthesis. Fe_dp/CMS, on the other hand, contained Fe^0^, Fe^2+^, and Fe^3+^ species, smaller Fe particles, and exhibited lower intrinsic activity. This suggests that the cocasting synthesis route promoted the transformation of the Fe precursor into more reduced Fe species, enhancing Fe mobility, leading to larger particle formation during pyrolysis. The higher intrinsic activity of Fe_cc/CMS is consistent with the structure-sensitive nature of NH_3_ synthesis, where larger particles are found to be more active.^39,41,42^ However, because Fe particle size, dispersion, Fe loading, oxidation state, and Fe-CMS interactions covary across these catalysts and may also impact activity, particle size cannot be independently established as the sole cause of the enhanced TOF. Particle size is therefore treated as a structural descriptor associated with the observed activity trends, rather than as the sole determinant of the higher TOF. It is also noted that the oxidation states of Fe are expected to change from the *ex situ* measured states under reaction conditions, but the measured differences are informative in understanding the differences in the initial Fe structures among the Fe/CMS samples. Post-reaction XPS analysis similarly showed mixed oxidized Fe species for all Fe/CMS samples (SI Figure 11).

**Figure 7:**
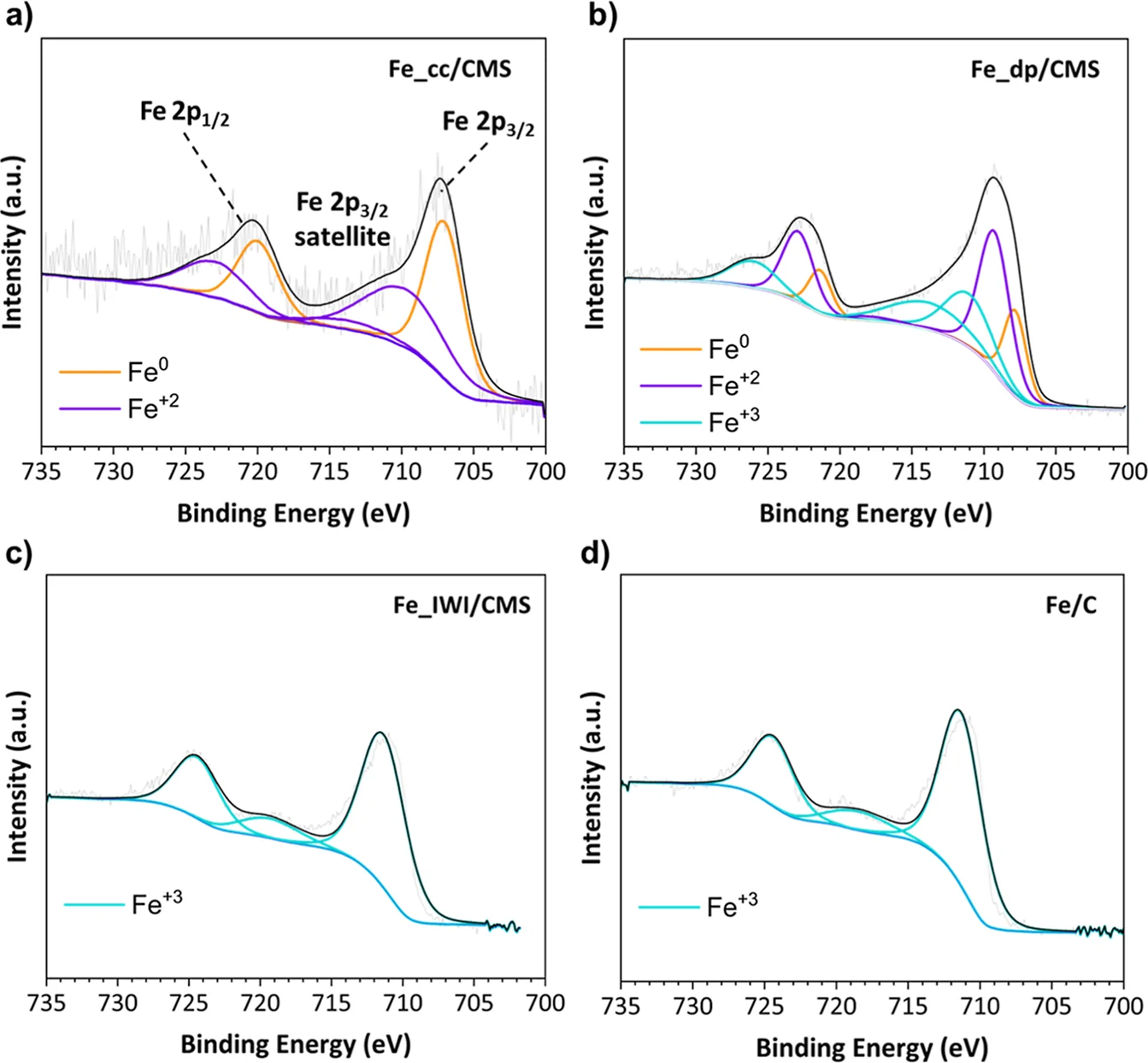
Fitted Fe 2p spectra of fresh samples of (a) Fe_cc/CMS, (b) Fe_dp/CMS, (c) Fe_IWI/CMS, and (d) Fe/C.

### 3.3 Effects of Fe Incorporation on the Morphology of the CMS Membrane

In this section, we discuss the results obtained from CO_2_ physisorption, Raman spectroscopy, and XRD to understand the broader morphological impact of Fe on the CMS membranes. The characterization results described earlier demonstrate that the Fe nanoparticles incorporated within CMS via various strategies alter their pore architecture, the ordering of carbon domains, and the overall structural properties.

As shown in Figure 8a, the pore size distributions of the prepared CMS membranes were characterized by CO_2_ physisorption at 0 °C using a density functional theory slit-pore model. All CMS membranes (with and without metals) show a bimodal distribution, with two distinct pore size regions corresponding to ultramicropores and micropores, a characteristic typical of CMS materials derived from polyimide precursors and attributable to microstructural rearrangements during pyrolysis.^11–13,44,45^ In the ultramicropore range (0.4–0.7 nm), the metal-free CMS membranes display the highest pore volume.^46^ Both Fe_cc/CMS and Fe_dp/CMS show reductions in ultramicropore volumes, with Fe_dp/CMS showing a more significant reduction. This is reflected in the NH_3_ permeation performance of the various samples, where Fe_dp/CMS shows the lowest NH_3_ permeability among the three samples—metal-free CMS, Fe_dp/CMS, and Fe_cc/CMS membranes. Similar sorption performance for the three samples, however, indicates negligible changes to the overall micropore capacities of these membranes.

**Figure 8:**
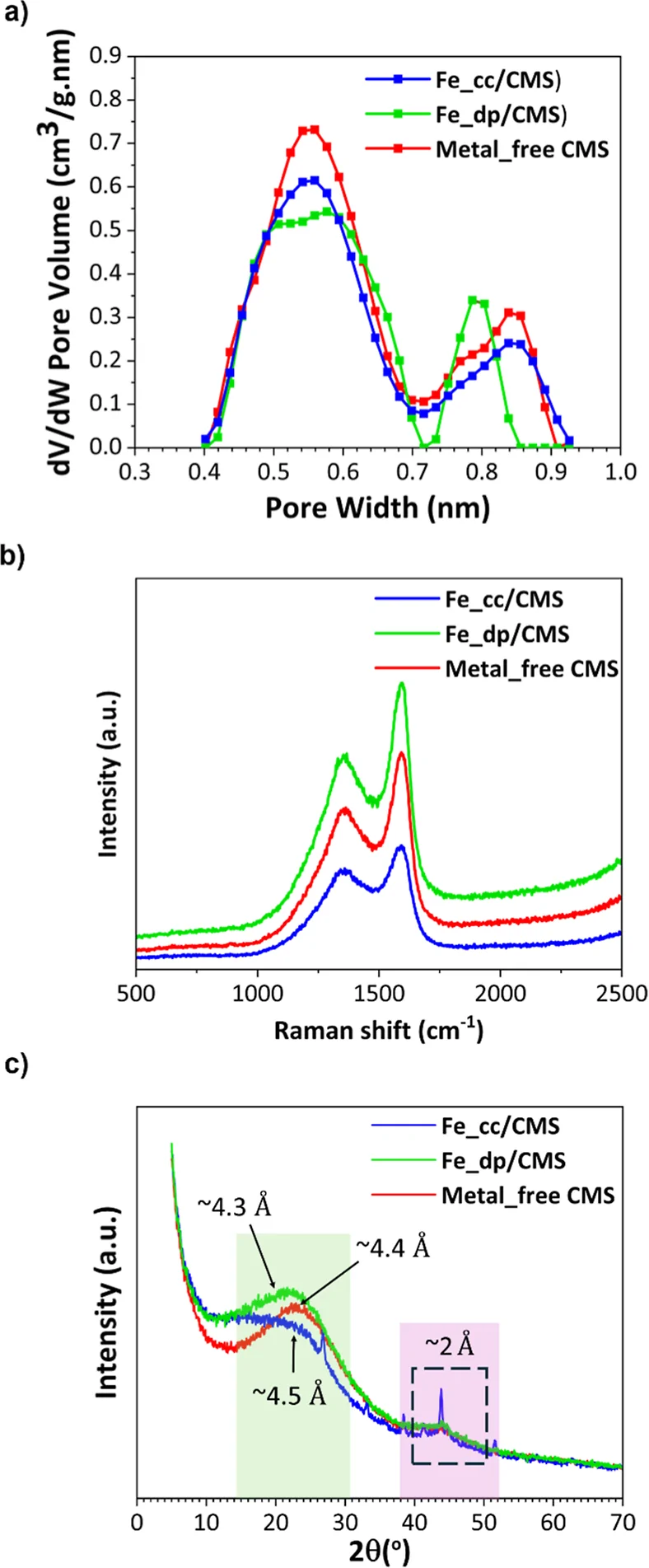
a) Pore size distributions obtained from CO_2_ physisorption at 0 °C, b) Raman spectra, and c) XRD spectra for metal_free, Fe_cc/CMS, and Fe_dp/CMS.

Raman spectroscopy and XRD demonstrate that all three CMS membranes are highly disordered amorphous materials. The two broad peaks can be captured in the Raman spectrum (Figure 8b) of each sample at ∼1350 cm^–1^ (D-band) and 1580 cm^–1^ (G-band). The D-band is associated with structural disorder arising from sp^3^ (diamond-like) bonds, while G-band corresponds to the in-plane stretching “ordered structure” for the sp^2^ (graphene-like) bond.^47^ Here, the relative degree of order and disorder in the three CMS membranes is evaluated by the ratio of *I*_D_/*I*_G_, shown in Table 6. The ratios of *I*_D_/*I*_G_ intensities show only modest variation between the metal free, Fe_cc, and Fe_dp/CMS membranes, indicating that Fe incorporation does not significantly alter the overall degree of graphitic ordering. These observed results are consistent with the previous studies and further confirm that the CMS membranes retain an amorphous carbon structure after Fe incorporation.^43,48–50^

**Table 6:** *I*_D_/*I*_G_ Ratios for Three Fresh CMS Membranes

samples	*I*_D_/*I*_G_
metal_free CMS	0.74
Fe_dp/CMS	0.75
Fe_cc/CMS	0.83

Similarly, the XRD patterns (Figure 8c) show broad amorphous peaks for three CMS samples, with an interlayer-like *d*-spacing (Bragg’s law) of (0.43–0.45 nm) for all samples. The Fe_cc/CMS membrane shows peaks corresponding to Fe phases, including Fe_*x*_O_*x*_ and Fe_*x*_C_*x*_, while these peaks are not observed for Fe_dp/CMS. Here, XRD and Raman spectroscopy were used to assess whether Fe incorporation altered the original amorphous carbon structure of the CMS membranes. The lack of substantial differences among the metal-free, Fe_cc/CMS, and Fe_dp/CMS membranes indicates that Fe incorporation largely preserves the overall CMS amorphous carbon structure. However, CO_2_ physisorption, permeation, and sorption isotherms provide insights into how Fe particles influence pore characteristics.

### 3.4 Discussion

Prior studies have demonstrated metal incorporation as a strategy to modify CMS membrane separation properties, and such studies have typically included a wide variety of metals such as Fe, Zn, Pd, Ag, Mg, Cu, Ca, and Si.^23–29,51^ However, these studies did not employ these metal-embedded CMS membranes as reactive platforms, and thus the geometric and electronic structure (effective size, dispersion, oxidation state, etc.) of the incorporated metal has not been previously analyzed. Furthermore, since most prior work was focused on separation alone, the effects of high-temperature exposure on the resulting metal structure were not analyzed. As a result, the synergistic impacts of these factors on the separation and catalytic performance of these CMS membranes have been open questions. Here, we have described first steps to answer these open questions about structure and performance in metal-embedded catalytic membranes. This work combines insights from the fields of heterogeneous catalysis and membrane science to develop a novel Fe-embedded CMS material active for NH_3_ synthesis and a candidate for future implementation into a membrane reactor. It was demonstrated that a synthesis strategy such as cocasting leads to twice as large particle size as compared to dip-coating or incipient wetness methods, and such size effects impact the overall NH_3_ production rates. On the other hand, the Fe_dp/CMS membranes have lower ultramicropore volumes vs the Fe_cc/CMS membranes, which result in low NH_3_ permeabilities and diffusion coefficients. Therefore, the trade-off exits in terms of the metal loading, where higher metal loading could lead to increased NH_3_ production (per g_catalyst_) but will be associated with reduced permeabilities and greater variability. These novel insights will guide future CMS membrane material design for diverse membrane reactor applications.

## 4 Conclusions

In this study, we investigated the dual-functional nature of Fe-embedded CMS (Fe/CMS) membranes and demonstrate that these materials are active for NH_3_ synthesis. Importantly, Fe/CMS maintain stable catalytic activity over prolonged time on stream while withstanding high temperature and pressure reaction conditions, demonstrating their potential for integration into a catalytic membrane reactor architecture. Sorption analysis, when complemented by CO_2_ physisorption measurements, revealed that all synthesis protocols resulted in a decline in the ultramicropore volumes to varying extents, while preserving the total micropore capacity. Permeation and diffusion estimates also point toward reduced ultramicropore dimensions in the Fe/CMS membranes, with Fe_dp/CMS having the smallest ultramicropores and therefore the lowest NH_3_ permeabilities and diffusion. We observe from electron microscopy that the different synthesis techniques used, dip-coating (Fe_dp/CMS), cocasting (Fe_cc/CMS), and incipient wetness impregnation (Fe_IWI/CMS), affect the particle size distribution within the CMS matrix. The particle size analysis and TOF calculations reveal that Fe_cc/CMS, with the largest particle size, showed the highest intrinsic activity among the set of Fe/CMS. This observed behavior is consistent with the structure-sensitive nature of Fe-catalyzed NH_3_ synthesis. Furthermore, XPS reveals that Fe incorporation strategies modulate the oxidation state of Fe within the CMS membranes—cocasting led to a mixture of Fe^0^ and Fe^2+^, while dip-coating led to a mixture of Fe^0^, Fe^2+^, and Fe^3+^. Further, Raman spectroscopy and XRD confirm that Fe incorporation does not alter the CMS structure significantly. Together, these findings reveal how the synthesis methods and the resulting metal and CMS properties govern the coupled relationship between membrane structure, NH_3_ transport, and the catalytic activity of these materials.

Building on these findings, future work will focus on three key directions. First, the effects of Fe loading will be investigated across the different CMS synthesis routes to determine the extent to which this parameter controls the catalytic activity and the separation performance of the resulting membranes. Second, membrane separation performance will be evaluated under NH_3_ synthesis conditions for a longer duration to demonstrate the stability of Fe/CMS membranes. As a practical way to integrate the metal-embedded CMS for the membrane reactor, direct measurements of NH_3_ permeability and NH_3_/H_2_ and NH_3_/N_2_ selectivities at elevated temperature and pressure, and preferably under mixed-gas conditions, should be performed. Finally, these promising Fe/CMS materials should be tested in a true membrane-reactor configuration, where NH_3_ synthesis and NH_3_ removal occur simultaneously. Together, these future studies will help establish design rules for catalytic CMS membrane reactors that balance the reactivity and separation properties.

## Supplementary Material



## Notes

Detailed experimental procedures; membrane and catalyst synthesis and pretreatment conditions; detailed characterization techniques; supplementary XPS, XRD, BET, and membrane characterization data; additional NH_3_ synthesis results; casting solution formulation; schematic of the pressure decay sorption system; CMS masking procedure; schematic of the constant volume permeation system; comparison of NH_3_ separation performance; NH_3_ permeation and diffusion coefficients; NH_3_ sorption isotherm regression; pore size distributions; NH_3_ production; comparison of NH_3_ production rates; NH_3_ production rates; Fe loaded in the CMS membrane; H_2_ chemisorption; deconvoluted spectra; deconvoluted C 1s peaks; comparison of sp^3^:sp^2^ ratios; and HR-TEM image (PDF)

## References

[1] Itoh N. A. (1987). Membrane
Reactor Using Palladium. AIChE J..

[2] Sirkar K. K., Shanbhag P. V., Kovvali A. S. (1999). Membrane
in a Reactor: A Functional
Perspective. Ind. Eng. Chem. Res..

[3] Champagnie A. M., Tsotsis T. T., Minet R. G., Webster A. I. (1990). A High Temperature
Catalytic Membrane Reactor for Ethane Dehydrogenation. Chem. Eng. Sci..

[4] Yousef
Motamedhashemi M. M., Egolfopoulos F., Tsotsis T. (2016). Flow-through Catalytic
Membrane Reactors for the Destruction of a Chemical Warfare Simulant:
Dynamic Performance Aspects. Catal. Today.

[5] Yousef
Motamedhashemi M. M., Monji M., Egolfopoulos F., Tsotsis T. (2015). A Hybrid Catalytic Membrane Reactor for Destruction
of a Chemical Warfare Simulant. J. Membr. Sci..

[6] Motamedhashemi M. M.
Y., Egolfopoulos F., Tsotsis T. (2011). Application of a Flow-through Catalytic
Membrane Reactor (FTCMR) for the Destruction of a Chemical Warfare
Simulant. J. Membr. Sci..

[7] Khajavi S., Jansen J. C., Kapteijn F. (2010). Performance
of Hydroxy Sodalite Membranes
as Absolute Water Selective Materials under Acidic and Basic Conditions. J. Membr. Sci..

[8] Yarema G., Yang J., Warty A., Liu Z., Hu L., Liu D. (2025). Membrane Reactors for Ammonia Production: Insights
into Ammonia-Separative
Membranes, Synthesis Catalysts, and Their Integration. ACS Nano.

[9] Adejumo M., Fazio N., Liguori S. (2025). NH3 Separation by ZnCl2 Immobilized
Molten Salt (IMS): Experimental and Modeling. J. Membr. Sci..

[10] Elimelech M., Zhu X., Childress A. E., Seungkwan H. (1997). Role of Membrane Surface Morphology
in Colloidal Fouling of Cellulose Acetate and Composite Aromatic Polyamide
Reverse Osmosis Membranes. J. Membr. Sci..

[11] Kim J., Kim S. S., Park M., Jang M. (2008). Effects of Precursor
Properties on the Preparation of Polyethylene Hollow Fiber Membranes
by Stretching. J. Membr. Sci..

[12] Shi L., Wang R., Cao Y., Liang D. T., Tay J. H. (2008). Effect
of Additives on the Fabrication of Poly­(Vinylidene Fluoride-*Co*-Hexafluropropylene) (PVDF-HFP) Asymmetric Microporous
Hollow Fiber Membranes. J. Membr. Sci..

[13] Kim S.-J., Yang S., Reddy G. K., Smirniotis P., Dong J. (2013). Zeolite Membrane Reactor for High-Temperature
Water-Gas Shift Reaction:
Effects of Membrane Properties and Operating Conditions. Energy Fuels.

[14] Arvanitis A., Sun X., Yang S., Damma D., Smirniotis P., Dong J. (2018). Approaching Complete CO Conversion and Total H2 Recovery for Water
Gas Shift Reaction in a High-Temperature and High-Pressure Zeolite
Membrane Reactor. J. Membr. Sci..

[15] Bang G., Moon D.-K., Kang J.-H., Han Y.-J., Kim K.-M., Lee C.-H. (2021). High-Purity Hydrogen
Production via a Water-Gas-Shift
Reaction in a Palladium-Copper Catalytic Membrane Reactor Integrated
with Pressure Swing Adsorption. Chem. Eng. J..

[16] Pera-Titus M., Llorens J., Cunill F., Mallada R., Santamaría J. (2005). Preparation
of Zeolite NaA Membranes on the Inner Side of Tubular Supports by
Means of a Controlled Seeding Technique. Catal.
Today.

[17] Ormerod R. M. (2003). Solid Oxide
Fuel Cells. Chem. Soc. Rev..

[18] Farrell B. L., Igenegbai V. O., Linic S. (2016). A Viewpoint on Direct Methane Conversion
to Ethane and Ethylene Using Oxidative Coupling on Solid Catalysts. ACS Catal..

[19] Masud M. A. A., Khuu N. H., Sanyal O., Tian Y. (2025). Advances in
Membrane-Assisted
Reactors: An Integrative Review for Modeling and Experiments. Sep. Purif. Technol..

[20] Xu L., Rungta M., Koros W. J. (2011). Matrimid®
Derived Carbon Molecular
Sieve Hollow Fiber Membranes for Ethylene/Ethane Separation. J. Membr. Sci..

[21] Steel K. M., Koros W. J. (2003). Investigation of Porosity of Carbon
Materials and Related
Effects on Gas Separation Properties. Carbon.

[22] Liu L., Bhowmick A., Cheng S., Blazquez B. H., Pan Y., Zhang J., Zhang Y., Shu Y., Tran D. T., Luo Y., Ierapetritou M., Zhang C., Liu D. (2023). Alkane Dehydrogenation
in Scalable and Electrifiable Carbon Membrane Reactor. Cell Rep. Phys. Sci..

[23] Wang Q., Huang F., Cornelius C. J., Fan Y. (2021). Carbon Molecular Sieve
Membranes Derived from Crosslinkable Polyimides for CO2/CH4 and C2H4/C2H6
Separations. J. Membr. Sci..

[24] Chu Y.-H., Yancey D., Xu L., Martinez M., Brayden M., Koros W. (2018). Iron-Containing Carbon
Molecular Sieve Membranes for Advanced Olefin/Paraffin
Separations. J. Membr. Sci..

[25] Barsema J. N., Balster J., Jordan V., Van Der
Vegt N. F. A., Wessling M. (2003). Functionalized Carbon Molecular Sieve
Membranes Containing
Ag-Nanoclusters. J. Membr. Sci..

[26] Lie J. A., Hägg M.-B. (2005). Carbon
Membranes from Cellulose and Metal Loaded Cellulose. Carbon.

[27] Hu L., Lee W.-I., Roy S., Subramanian A., Kisslinger K., Zhu L., Fan S., Hwang S., Bui V. T., Tran T., Zhang G., Ding Y., Ajayan P. M., Nam C.-Y., Lin H. (2024). Hierarchically Porous
and Single Zn Atom-Embedded Carbon Molecular Sieves for H2 Separations. Nat. Commun..

[28] Zhang J., Wang Z., Jin J. (2023). Carbon Molecular
Sieve Membranes
with a Rationally Designed Polymer Precursor for Improved Propane/Propylene
Separation. Ind. Eng. Chem. Res..

[29] Lin Y.-C., Zheng Z., Knibbe R., Li J.-Y., Tseng H.-H., Wiley D. E., Wang D. K. (2025). Ultramicroporous
Carbon Molecular
Sieve Membranes via Co-Pyrolysis of Cobalt-2,6-Bis­(2-Benzimidazolyl)
Pyridine and Polyetherimide for Enhanced Gas Separations. Carbon.

[30] Kulkarni M., Roy D., Khuu N. H., Sanyal O., Ball M. R. (2023). Expanding the Toolbox
for Development of Metal-based Dual-functional Catalytic Membranes. AIChE J..

[31] Adejumo M., Zabi A. M., Fazio N., Liguori S. (2026). NH_3_ Permeation
Performance of ZnCl_2_ -Immobilized Molten Salt Membranes
at Elevated Pressures. Ind. Eng. Chem. Res..

[32] Farsi, M. Ammonia Production from Syngas: Plant Design and Simulation. In Advances in Synthesis Gas: Methods, Technologies and Applications; Elsevier, 2023; pp 381–399. 10.1016/B978-0-323-91879-4.00012-6.

[33] Jazani O., Adejumo M., Elharati M. A., Liguori S. (2024). Low-Carbon Hydrogen
Production via Ethanol Reforming Reactions in Membrane Reactors: Recent
Advances and Future Directions. Energy Fuels.

[34] Kulkarni M., Robey E., Clutter B., Ball M. R. (2026). Investigation of
Molybdenum Iron Catalysts for Ethylene Production via Non-Oxidative
Coupling of Methane. ChemCatChem.

[35] Sanyal O., Hays S. S., León N. E., Guta Y. A., Itta A. K., Lively R. P., Koros W. J. (2020). A Self-Consistent
Model for Sorption
and Transport in Polyimide-Derived Carbon Molecular Sieve Gas Separation
Membranes. Angew. Chem., Int. Ed..

[36] Yoon Y. H., O’Nolan D., Beauvais M. L., Chapman K. W., Lively R. P. (2023). Direct
Evidence of the Ultramicroporous Structure of Carbon Molecular Sieves. Carbon.

[37] Ma Y., Jue M. L., Zhang F., Mathias R., Jang H. Y., Lively R. P. (2019). Creation of Well-Defined
“Mid-Sized”
Micropores in Carbon Molecular Sieve Membranes. Angew. Chem., Int. Ed..

[38] Somorjai G. A., Carrazza J. (1986). Structure Sensitivity of Catalytic Reactions. Ind. Eng. Chem. Fundam..

[39] Zhang B.-Y., Su H.-Y., Liu J.-X., Li W.-X. (2019). Interplay Between
Site Activity and Density of BCC Iron for Ammonia Synthesis Based
on First-Principles Theory. ChemCatChem.

[40] Grosvenor A. P., Kobe B. A., Biesinger M. C., McIntyre N. S. (2004). Investigation of
Multiplet Splitting of Fe 2p XPS Spectra and Bonding in Iron Compounds. Surf. Interface Anal..

[41] Spencer N. D., Schoonmaker R. C., Somorjai G. A. (1981). Structure Sensitivity in the Iron
Single-Crystal Catalysed Synthesis of Ammonia. Nature.

[42] Dumesic J. A., Topsøe H., Khammouma S., Boudart M. (1975). Surface Catalytic and
Magnetic Properties of Small Iron Particles: II. Structure Sensitivity
of Ammonia Synthesis. J. Catal..

[43] Liu Z., Qiu W., Quan W., Koros W. J. (2023). Advanced Carbon Molecular Sieve Membranes
Derived from Molecularly Engineered Cross-Linkable Copolyimide for
Gas Separations. Nat. Mater..

[44] Rungta M., Wenz G. B., Zhang C., Xu L., Qiu W., Adams J. S., Koros W. J. (2017). Carbon Molecular
Sieve Structure
Development and Membrane Performance Relationships. Carbon.

[45] Koros W. J., Zhang C. (2017). Materials for Next-Generation
Molecularly Selective Synthetic Membranes. Nat.
Mater..

[46] Leonard B., Khuu N. H., Mascaro A. S., Mize A., Bhattacharyya D., Sanyal O. (2026). Effects of Diamine
Based Modifications on the Morphology
and Separation Performance of Matrimid-Derived Carbon Molecular Sieve
(CMS) Hollow Fiber Membranes. J. Membr. Sci..

[47] Ferrari A. C., Robertson J. (2000). Interpretation of Raman Spectra of
Disordered and Amorphous
Carbon. Phys. Rev. B.

[48] Zheng Y., Miao G., Yang C., Huang J., Li X., Du S., Xiao J. (2026). Fast Size-Sieving
Separation of C2H4/C2H6 by Pore Engineering
of Scalable Carbon Molecular Sieve Granules. Chem. Eng. Sci..

[49] Liu Z., Qiu W., Quan W., Liu Y., Koros W. J. (2021). Fine-Tuned Thermally
Cross-Linkable 6FDA-Based Polyimide Membranes for Aggressive Natural
Gas Separation. J. Membr. Sci..

[50] Ghasemiestahbanati E., Yoon Y. H., Lively R. P., Shaibani M., Majumder M., Hill M. R. (2023). A Triple-Functional Carbon Molecular
Sieve (CMS) That
Addresses the Performance Trilemma in Practical Lithium Sulfur Batteries. Carbon.

[51] Zhao G., Wang K., Fang C., Wang Y., Wang D., Song Z., Lei L., Xu Z. (2024). Precise Molecular Sieving
Using Metal-doped Ultramicroporous Carbon Membranes for H_2_ Separation. AIChE J..

