# Supplementary material for: Effects of Fe Incorporation Strategies on the Coupled Catalytic and Transport Properties of Fe-Embedded Carbon Molecular Sieve Membranes
Source: ACS Mater Au. 2026 Oct 7;6(6):1566–78. doi: 10.1021/acsmaterialsau.6c00171 (PMC13760628; doi:10.1021/acsmaterialsau.6c00171)
Supplement: Supplementary file 1 [file mg6c00171_si_001.pdf]

Supplementary Information

Effects of Fe incorporation strategies on the coupled catalytic and transport properties of Fe-embedded carbon molecular sieve membranes

Nhan H. Khuu [a ⊥], Mihir Kulkarni [b ⊥], Majed Alam Abir[a], Antonio Aguirre[a], Trenton Rosenthal[a], Alyssa Mize[a], Joseph ZM Harrah[a], Madelyn R. Ball [b]*, and Oishi Sanyal[a]*.

[a] Department of Chemical and Biomedical Engineering, West Virginia University, 1306 Evansdale Dr, Morgantown, WV, 26506, USA.

[b] Department of Chemical Engineering and Materials Science, Michigan State University, 428 S Shaw Ln, East Lansing, MI, 48824, USA.

⊥ *N.H.K and M.K. contributed equally to this work*

*Corresponding author emails: mball@msu.edu, Oishi.sanyal@mail.wvu.edu

Materials and methods

A) Synthesis:

The polymer precursor 6FDA-DAM-DABA (3:2) (6FDA = 4,4'- (hexafluoroisopropylidene) diphthalic anhydride; DAM = 2,4,6- trimethyl-1,3-diaminobenzene; DABA = 3,5-diaminobenzoic acid) (Mw. 203K) was obtained from Akron Polymer Systems (Akron Polymer Systems, Ohio). Sure-seal bottles of Tetrahydrofuran (THF) (99.9% purity), Methanol (99.9% purity) were purchased from Sigma-Aldrich (St Louis, MO). Pure-component Argon gas (UHP quality) was obtained from Airgas (Morgantown, WV). Pure-component H_2, and N_2 gas (UHP quality) were purchased from Matheson (Fairmont, WV). Pure-component NH_3 gas (research grade quality) was purchased from Butler Gas (Morgantown, WV). Iron (II) acetylacetonate ($Fe(acac)_2$) (99.95% purity) was purchased from Erez tech (Johns Creek, GA). Hydrochloric acid (HCl) (# A144C212) was purchased from Fisher Scientific (Canada). Nitric acid-ACS reagent 70% (HNO_3) (# 7697-37-2) was purchased from Sigma Aldrich. The chemicals used for cleaning of equipment were acetone (20L drum ACS grade 99.5% purity), purchased from Sigma-Aldrich (Sheboygan Falls, WI) and 1-Methyl-2-pyrrolidinone (NMP) (20L drum ACS grade 99% purity) purchased from Thermo Scientific. The polytetrafluorethylene (PTFE) 0.22 µm filter (VWR International, #76479-006, Radnor, PA) and (PTFE) 0.45 µm (VWR International, #76479-004, Randor, PA) were obtained from VWR International LCC.

To synthesize the conventional catalyst, Fe/C, activated carbon (AC) support (NORIT RX3 Extra), 70% HNO_3 (ACS grade) nitric acid to pretreat the activated carbon, and the iron precursor, iron (II) nitrate nonahydrate ($Fe(NO_3)_3 \cdot 9H_2O$, 99.95% purity, were obtained from Sigma-Aldrich. Industrial grade nitrogen was used to calcine the Fe/C catalyst.

i) Fe embedded carbon molecular sieve membranes (Fe/CMS) synthesis

Table S1. Casting solution formulation for metal-containing polymer dense films and metal-free polymer dense films

Vial composition	Solvent	Solute
A	20mL THF	~0.5g polymer precursor
B	5mL THF	~0.08g $Fe(acac)_2$
C	40mL Methanol	~3.6g $Fe(acac)_2$
D	20ml THF	1.16g polymer precursor
E	20mL THF	1g polymer precursor
F	5mL THF	~0.03g $Fe(acac)_2$

a) Formation of metal free polymer dense film membranes:

In this work, the 6FDA-DAM-DABA (3:2) polyimide was used as a precursor to synthesize carbon molecular sieve membranes. The precursor powder was dried under vacuum (Edwards Vacuum LLC, #A73602983, West Sanborn, NY) at 120 ˚C for 24 hours to remove moisture. The dried polymer powder was dissolved in 20 mL of THF in a 40mL vial (vial A,D, and E, **Table S1**) to form a polymer solution of a specific wt.%, and the solution was then placed on a rolling mixer overnight to ensure a completely homogenous solution. All the polymer vials were filtered using a 0.22 µm (PTFE) filter to remove any solid impurities prior to casting or mixing with metal precursors.

The polymer film was casted inside a glove bag (Cole-Parmer, #EW-04408-52, Vernon Hills, IL), that was inflated with inert N_2 three times. Next, a 60 mL jar of THF was placed inside the glove bag for at least 3 hours to maintain a THF-rich atmosphere. The glove bag was kept in a fume

hood for the entire process. The polymer solution was then poured on a Teflon dish inside the glove bag, and followed by covering with glassware to ensure uniform evaporation. After ~72 hours, the defect-free polymer film was removed from the glove bag and dried under vacuum at 120 ˚C for 24 hours to remove residual solvent.

For permeation tests, a 7 wt.% solution of polymer (vial D, **Table S1**) was made, while 3 wt.% (vial A, **Table S1**) solution was used for the reaction and sorption experiments and all material characterizations such as X-ray diffraction (XRD), X-ray photoelectron spectroscopy (XPS), Raman spectroscopy, BET CO_2 physisorption, High-resolution transmission electron microscopy (HR-TEM), H_2 chemisorption and Inductively coupled plasma optical emission spectrometry (ICP-OES). Since carbon films tend to be brittle, a high wt.% yielded a thicker film with good mechanical stability for permeation experiments. A circle hollow punch (3/4") was used to cut 3 or 4 membrane samples out of the whole polymer membrane prior to pyrolysis.

b) Formation of Fe co-casting (Fe_cc) polymer dense film membranes:

The Fe(acac)$_2$ was dissolved in 5 mL THF in a 20 mL vial (vial B, and F, **Table S1**) to form a Fe-containing solution and then placed on a rolling mixer overnight for complete Fe dissolution. The Fe co-casting solutions were filtered using a 0.45 µm PTFE filter to remove impurities from the solutions prior to mix with polymer vials.

The Fe co-casting solution was added to vial A (**Table S1**) and mixed by a vortex mixer to ensure well-mixed solution .The casting method followed the same procedure as that used for the metal free polymer dense film membranes. After drying at 120 ˚C for 24 hours, the Fe co-cast membrane exhibited an orange-color. The maximum Fe(acac)$_2$ that could be incorporated in Fe_cc

membrane was ~0.07 g. Beyond this amount, defects such as gel formation, membrane curvature, and bubbling were observed.

For the permeation tests, vials E+B (**Table S1**), and vials E+F (**Table S1**) were made, while (3 wt.%) vials A+B (**Table S1**) were used for the reaction and sorption experiments, and all characterizations. A circle hollow punch was used to cut 3 to 4 circle membranes out of a whole membrane prior to pyrolysis.

c) Formation of Fe dip_coating (Fe_dp) polymer dense film membranes:

~3.6 g of $Fe(acac)_2$ was dissolved in 40 mL methanol in a 60 mL vial (vial C, **Table S1**) to form a Fe-containing solution and placed on a rolling mixer overnight to ensure a completely homogenous solution.

For this method, the metal free polymer membranes were dipped into the Fe dip-coating solutions (Vial C, **Table S1**) within the glove bag under N_2 atmosphere (inflated with N_2 three times) saturated with 60 mL methanol. Following a minimum exposure time of 36 hours, the membranes were taken out, rinsed thoroughly with methanol, and dried under vacuum at 120 ˚C for 24 hours, resulting in a Fe_dp polymer. The resulting membrane samples were also orange in color, similar to the Fe_cc membranes.

For the permeation tests, the polymer was formed from a 5-6 wt.% polymer solution (vial E, **Table S1**), while 3 wt.% solution was used for the reaction and sorption experiments and all material characterizations. First, the polymer solution (vial E) was cast, then the membrane was dipped into the $Fe(acac)_2$ solution (vial C) (**Table S1**) to form the Fe_dp membrane. A circle hollow punch (3/4”) was used to cut 3 to 4 circle membranes out of a whole membrane before pyrolysis.

d) Fe CMS membranes via Incipient Wetness Impregnation (Fe_IWI/CMS):

In this process, a metal free CMS was crushed into a fine powder. The wetness point, or quantity of solvent to completely fill the CMS pores, was measured and determined to be 1.484 g of methanol/g of CMS. The IWI CMS has a theoretical Fe loading of 3wt.%, whereby the desired amount of $Fe(acac)_2$ was dissolved in the methanol according to CMS wetness point to synthesize 2-3wt.% CMS membrane. The Fe solution was added dropwise to the CMS, mixing well between each addition. The Fe_IWI/CMS was transferred to a ceramic crucible and dried in static air at 120 ˚C for 30 minutes. The dried sample was then crushed into a fine powder using mortar and pestle and calcined under N_2 (50 sccm) at 500 ˚C (ramp rate of 3.5 ˚C/min) for 4 hours, followed by cooling under N_2 to room temperature.

e) Synthesis of CMS dense films (metal free and metal containing) via high temperature pyrolysis

The pyrolysis process was performed in a three-zone furnace with an accompanying controller (Thermcraft Inc, # XST-3-0-24-3V2-F01, Winston-Salem, NC) to achieve a uniform temperature in the system. The dense membranes were placed on a quartz plate inside a quartz tube (MTI, Richmond, CA; 2.36” OD x 2.16” ID x 40” L). The ends of the quartz tube were sealed with metal flanges (MTI, Richmond, CA # FL60R) with a silicone O-ring (MTI, Richmond, CA, #SOR-60). The pyrolysis experiments were carried out under an inert atmosphere (UHP Argon) under a constant flow of 700cc (STP)/min. The gas flow was controlled using a mass flow controller (Alicat Scientific, Tucson, AZ # 253237). Ar purging was conducted for at least 8 hours to reduce the O_2 level to below 1 ppm and the O_2 concentration was monitored by an oxygen analyzer (CEA Instruments Inc, Westwood, NJ, Rapidox 2100 series). The polymer dense film membranes (with and without metal) were pyrolyzed at 550 ˚C under UHP Argon to form CMS dense film membranes using a pyrolysis protocol reported in **Figure 1 e)**.

A cleaning protocol was conducted between successive pyrolysis runs to ensure no residue from previous runs:

1) Metal flanges, oxygen sensor block, and all Swagelok® fittings connection were soaked in hot NMP (~110 ˚C) overnight.
2) Then the parts were rinsed and allowed to soak in DI water for 6 hours to ensure all NMP removal from the parts.
3) Next, the parts were soaked in acetone for ~4 hours at room temperature.
4) Finally, all the parts were dried under vacuum at 120 ˚C for 30 mins before reassembling.

The quartz tube and the glass plate were cleaned with Dawn dish soap and rinsed with acetone prior to reassembly with all the connections. This was followed by a bake-out protocol (900 ˚C for 8 hours under 900 cc (STP) air/min) to remove any residue that could potentially contaminate the samples. After the bake-out, both the quartz tube and quartz plate were rinsed with acetone one more time before pyrolyzing the membrane samples. All these steps are required to ensure no cross-contamination occurred between metal free and metal containing samples.

Conventional Fe catalyst (Fe/C)

The activated carbon (AC) (NORIT RX3 Extra, Sigma Aldrich) support was pretreated prior to the synthesis of iron catalysts. AC was stirred overnight in a 4M nitric acid solution (prepared using 70% HNO_3, ACS grade), filtered, washed with DI water, and then dried in static air at 100 ˚C for 15 hours. Fe catalysts supported on activated carbon were synthesized using incipient wetness impregnation (IWI). The necessary quantity of the Fe precursor was dissolved in a known volume

of 18 MΩ water, determined by measuring the wetness of the carbon support (similar to the Fe_IWI CMS membranes). This solution was then added dropwise to the carbon support and mixed until uniform. Then, the catalyst was covered with aluminum foil and air dried overnight at room temperature followed by drying at 110 ˚C in static air overnight. The catalyst was calcined at 500 °C for 4 hours under 50 mL/min nitrogen (Industrial, Airgas). The prepared 20 wt.% iron catalyst is labeled as 'Fe/C'.

B) Performance of Fe/CMS

a. Reactivity

Prior to the reactivity tests, membrane samples were stored in petri dishes that were placed in a vacuum desiccator or glove box. The membrane samples were ground using a mortar and pestle before mixing with silicon carbide (SiC, Strem Chemicals, 100 mesh). Catalytic activity measurements were performed in a stainless-steel tubular reactor with a 0.375" OD and 0.035" wall thickness. The catalyst bed was comprised of the Fe/C catalyst or the membrane samples (metal_free CMS, Fe_cc CMS, Fe_dp CMS and Fe_IWI CMS) (~0.1 – 0.5 g) diluted with SiC at approximately 3 times the sample weight. The catalyst or membrane and silicon carbide mixture was supported between plugs of quartz wool (Technical Glass Products). A K-type thermocouple (Omega) was inserted from the top of the reactor ensuring its tip touched the top of the catalyst bed. A vertical tube furnace (Mellen Co. Inc.) was used to heat the reactor. The desired temperature inside the reactor tube was controlled by a temperature controller (Dwyer Instruments) connected to the tubular furnace. H_2 (UHP), and N_2 (Industrial) were purchased from Airgas and used without further purification. These gases were controlled using Bronkhorst 'EL-

FLOW Prestige' mass flow controllers capable of maximum operating pressure of 1500 psi using the FlowSuite software. The catalysts and membrane samples were pretreated *in situ* under a stoichiometric mixture of H_2:N_2 (3:1) at a total flow rate of 30 ml/min. This gas feed was kept constant throughout the duration of the experiment. The samples were first pretreated at 480 °C (heating at 4 °C/min) for 6 hours and then the temperature was increased to the reaction temperature of 500 °C (2 °C/min). Once the temperature reached 500 °C, the reactor was pressurized to 400 psi using a downstream back pressure regulator (Swagelok). The pressure readings before and after the catalyst bed were measured using two pressure gauges (Swagelok, 40 – 1000 psi). Typically, around 10 psi pressure drop was observed across the reactor assembly. Once the reaction pressure stabilized at 400 psi, the reaction sequence started after a 10-minute flush time. A three-way valve was located downstream of the reactor to connect the outlet flow to either a bubble-flow meter or the gas chromatography system and vent. A second back pressure regulator was positioned on the vent side to maintain constant pressure to the GC. Gas composition quantification was conducted on an Agilent 990 Micro GC gas chromatography system equipped with a three-channel setup. The first channel was equipped with a Molesieve 5Å column with a backflush along with a CP-PORABOND column to quantify N_2 and H_2 using Ar as carrier gas. The second channel was equipped with a CP-Volamine column for NH_3 detection using H_2 as the carrier gas. Both channels are connected to thermal conductivity detectors (TCD). Agilent OpenLab CDS software was used for data acquisition and analysis. N_2 (Industrial, Airgas), H_2 (UHP, Airgas) and 5 mol.% NH_3/Ar (Airgas) gases were used to calibrate the GC. Typically, the experiment was run for a minimum of 20 hours time on stream or until steady state was achieved.

b. Sorption measurements

The equilibrium sorption isotherms for CMS membranes dense films (metal-free, Fe_cc and Fe_dp) were measured using a pressure decay sorption setup reported in prior publications (Figure S1)[1]. For the sorption isotherms, two replicates per sample were performed. The CMS membranes were crushed into small pieces by a mortar and pestle, then wrapped with aluminum foil to hold the sample securely in the cell chamber. The weight of CMS samples with and without the aluminum foil was recorded prior to the experiments. The entire setup was immersed in a temperature-controlled water bath maintained at 35 °C, regulated by a temperature controller (Sirman, Italy # 69060058). The system was evacuated under vacuum for a minimum of 12 hours to remove residual gases. Before introducing the gas of interest to the system, valve D was fully closed to isolate the cell chamber, then gas was fed to the reservoir chamber, and valve C was closed. The gas was kept in the reservoir for 10 minutes of thermal equilibration, then valve D was opened to introduce the gas to the cell chamber. NH_3 is highly condensable at 35 °C and requires more than 3 hours to reach steady state for each pressure point. The decline in cell chamber pressure over time, resulting from gas sorption by the membrane, was monitored using a digital pressure transducer (Omega Engineering, Inc., Norwalk, CT, # PX409-500AUSBH). The sorbed gas by the membrane was calculated based on mole balance equation[1].

$$n_{total} = n^{i}_{reservoir} + n^{i}_{cell} = n^{f}_{reservoir} + n^{f}_{cell} + n^{f}_{sample} \qquad \text{(Equation 1)}$$

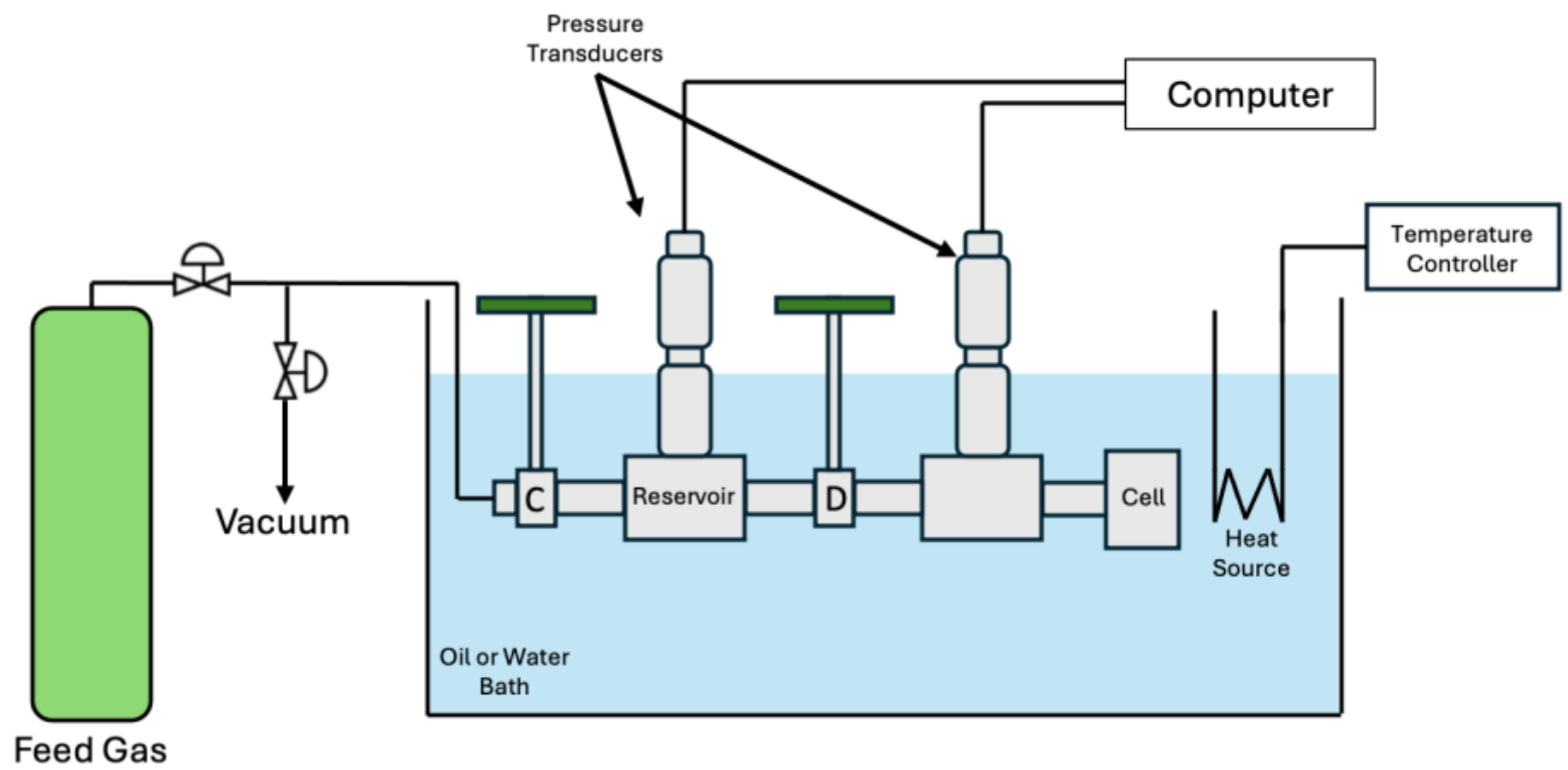

Figure S1. Schematic of the pressure decay sorption system

Equilibrium pressures were plotted as a function of sorbed gas concentration and analyzed using the dual-mode sorption model[2] (Equation 2). Sorption parameters were extracted using this model to determine both Henry's law and Langmuir-type contributions for all membrane samples.

The pure sorption coefficient is defined as the penetrant concentration of A (C_A) sorbed by the material at the equilibrium partial fugacity (f_A).

$$S_A = \frac{C_A}{p_A} = k_{C,A} \times f_A + \frac{C'_{L,A} \times b_A}{1+b_A \times f_A} \qquad \text{(Equation 2)}$$

Where $C'_{L,A}$ is the Langmuir coefficient, b_A is the Langmuir affinity, and $k_{C,A}$ is the continous mode's sorption constant at equilibrium fugacity (f_A), where $f_A = \text{partial pressure } (p_A) \times$ $\text{fugacity coefficient } (\emptyset_A)$. As NH_3 is the highly condensable gas at the testing conditions, $\emptyset_A$ varies with pressure. $\emptyset_A$ were calculated for each gas using CHEMCAD.

c. Permeation measurements

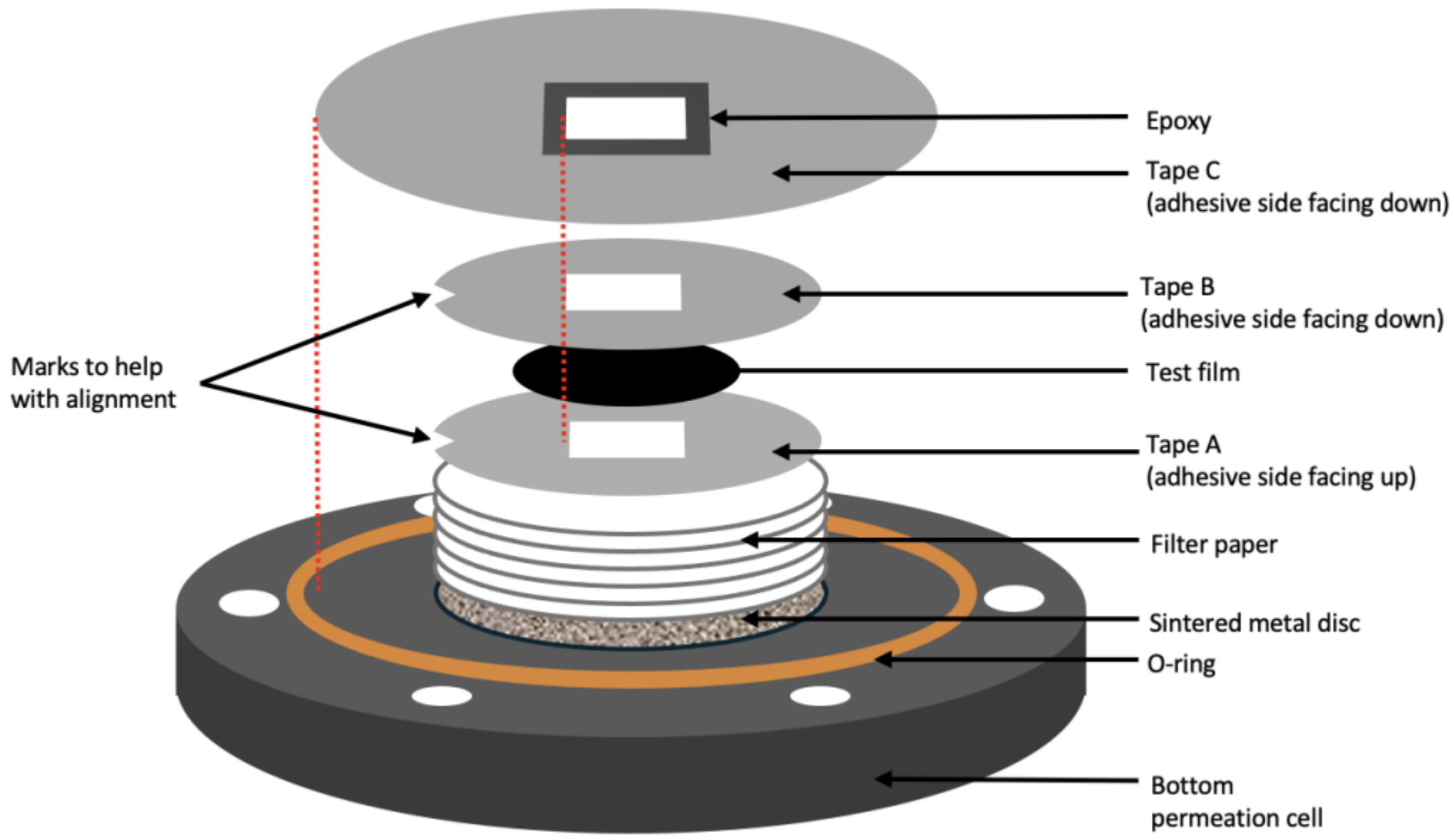

Figure S2. CMS masking procedure for permeation testing. Figure was adapted and modified from [3]

The CMS dense film permeation cell was measured in a constant volume permeation system at 35 ˚C, the details of which are described below. A square permeation area (~ 0.4 cm x 0.4 cm) was cut at the center of the impermeable aluminum tapes prior to attaching them to the permeation cell. The CMS dense film membranes were masked between two pieces of impermeable aluminum tape, followed by six layers of filter paper (Whatman Inc, Piscataway, NJ, Cat # 1001-042) between the masked film and the base of the cell to provide a cushion when testing, as described in **Figure S2**. A larger impermeable aluminum tape was placed on top of the masked film and a well-mixed Epoxy (Devcon Inc, USA, #1420) was applied at the interface edges between the film and the tape for sealing purposes. The masked film was kept in a low humid environment

(< 25%) and 25 ˚C for epoxy curing for at least 24 hours before assembling the full cell with O-ring and screws for permeation measurements.

The pure gas permeation measurements were performed at 30 psi (upstream) and vacuum (Edwards Vacuum LLC, West Sanborn, NY, #A73602983) (downstream) at 35 ˚C for NH_3, wherein an operating permeation system steps were made to reduce the membrane breakage, as listed in below to ensure the integrity of the CMS dense film membranes. For safety purposes, NH_3 tank was kept in gas cabinet, and an NH_3 detector with USA NIST calibration (Forensics Detectors, CA, USA, # FD-90A-NH_3) was used to detect a potential NH_3 leak. The temperature controller was set up with 1/16 DIN controller using on/off default function (Newark Company, USA, #CN742), T type thermocouple (Newark Company, USA, #SA3-T), and two 5"x 5" flexible silicone heaters (Newark Company, USA, #SRFGA-505/5) to maintain the temperature during the experiment.

The permeability of the dense film membranes was calculated using Eq3.

$$P = \frac{1.764\times10^{6}\times V\times l}{(T+273.15)\times A\times \Delta p} \times \left(\frac{dp}{dt}\right) \qquad \text{(Equation 3)}$$

where, P is the Permeability (Barrer), V is the downstream volume (cm^3), l is the membrane thickness (mil), $\left(\frac{dp}{dt}\right)$ is the downstream pressure rising rate (Torr/s), A is the area of the membrane for permeation (cm^2), and Δp is the partial pressure difference across the membrane (psi).

The membrane area and membrane thickness were measured by a digital microscope (Dunwell Tech, Inc., Torrance, CA, #AF7515MZT). The method for using the constant volume permeation system (**Figure S3**) is described below:

Operating permeation system steps**:**

1) Gas tank and valve 1 are opened at the same time.

2) The pressure is increased to 15psi which shows up on the digital pressure transducer (Omega Engineering, Inc., #PX409-500GUSBH, Norwalk, CT).
3) Valve 4 is slowly opened to introduce a gas to the permeation cell until it is fully opened, then fully closed it again.
4) Valve 6 is gradually opened until the vacuum pressure transducer (MKS Instruments, Inc., #631F12T8FPAB, Andover, MA) reads below <1 torr, then fully closed it again.
5) Valve 4 is slowly reopened and observed if the pressure increases rapidly. If it rises immediately to 100 torr, the membrane is broken.
6) Valve 6 is slowly opened again until it reaches below 0.5 torr, and next fully closed it again. If torr vacuum unit went up slowly, it means the membrane works well.

The pressure is increased to 30 psi and steps 3 to 7 are repeated.

In this paper, permeability is reported in terms of the unit, Barrer.

$$1\ Barrer = 10^{-10}\frac{cc(STP)\times cm}{cm^2\times s\times cm.Hg} \quad \text{(Equation 4)}$$

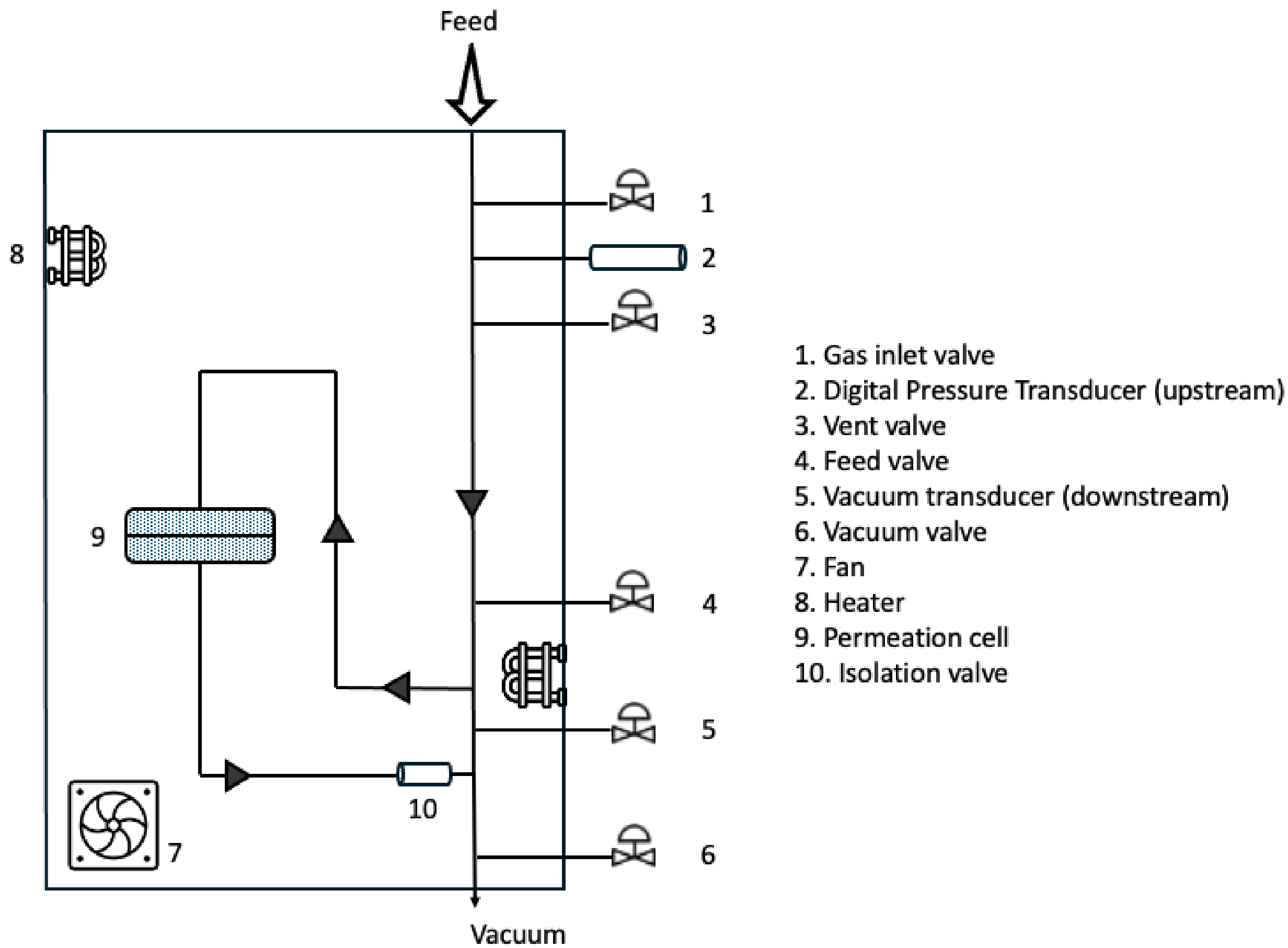

Figure S3. Schematic of constant volume permeation system

C) <u>Materials characterization</u>:

a) <u>Inductively coupled plasma optical emission spectrometry (ICP-OES)</u>:

Aqua regia (15 mL HCl (concentrated, Fisher Scientific) and 5 mL HNO_3 (concentrated, Sigma-Aldrich)) were added to approximately 20 mg of ground Fe_cc/CMS or Fe_dp/CMS membrane sample in a beaker. The beaker was covered with parafilm, and the solution was kept at room temperature for 24 hours. The solution was diluted with deionized water to 25 mL and filtered via gravity filtration using Whatman Grade 1 filter papers, followed by dilution to a final volume of 50 mL. The 50 mL solution was directly analyzed using Perkin Elmer Avio 550 Max ICP-OES.

The Fe_IWI/CMS, Fe_cc/CMS, and Fe_dp/CMS sample was analyzed on Agilent 5800 ICP-OES after digesting in a CEM Mar6 microwave digestion system. Approximately 30 mg of ground Fe_IWI/CMS sample, 5 mL HNO_3 (concentrated, Fisher Chemical), and 5 mL HCl (concentrated, Fisher Chemical) were added to a digestion vessel. The default 'Carbon' program with the following steps was used: the temperature was ramped to 210 ˚C in 29 minutes and then maintained for 30 minutes. Then, the vessels were slowly cooled down below 65 ˚C and let stand for another 30 minutes. Upon cooling, the contents of the vessel were transferred to a 50 mL centrifuge tube. 18 MΩ H_2O was used to dilute the digested solution and then a 1 mL aliquot was taken from this solution in another centrifuge tube. The aliquot was further diluted to a final volume of 10 mL using 18 MΩ H_2O.

b) X-ray diffraction (XRD):

PANalytical X'Pert Pro X-ray Diffractometer with a Cu-Kα source powered at 45 kV and 40 mA was used to obtain the X-ray diffraction (XRD) patterns of the CMS membranes. The data was recorded in the 2Θ scan range of 5° to 70° with a scan step size of 0.03°. HighScore Plus software was used to analyze the acquired data.

c) X-ray photoelectron spectroscopy (XPS):

X-ray photoelectron spectra of the Fe_cc/CMS and Fe_dp/CMS membrane samples were collected using Physical Electronics PHI 5000 VersaProbe X-ray Photoelectron Spectroscope equipped with a monochromatic Al Kα (1486.7 eV) X-ray source, spot size of 100.0 µm, 23.1 W power and emission angle of 46.4°. Survey scans were conducted at a pass energy of 117.4 eV, and the detailed scan for Fe2p at 23.5 eV. Spectra for Fe_IWI/CMS and Fe/C were collected on a

Kratos Axis Supra+ X-ray Photoelectron Spectrometer with an Al Kα (1486.7 eV) X-ray source. Survey scans were conducted at a pass energy of 160 eV, while the detailed scans for Fe2p at were conducted at 20 eV (electron current of 15 mA for Fe/C and 30 mA for Fe_IWI/CMS). The post-reaction analysis was performed approximately six months after the samples were tested under reaction conditions. During this period, the samples had been exposed to air and remained mixed with quartz wool and SiC from the reactor bed. These factors likely contributed to oxidation of the Fe surface and dilution of the Fe signal, resulting in the comparatively low Fe 2p intensity and limiting quantitative interpretation of the post-reaction oxidation-state distribution. CasaXPS software was used to process and fit the spectra, and the spectrum shift calibration was performed using C 1s at 284.8 eV.

d) CO_2 physisorption:

The CMS samples were degassed overnight at 120 °C under vacuum (~5 mmHg) to remove any contaminants prior to running the physisorption measurements. Porosity measurements were performed using CO2 physisorption at 0 °C on a Micromeritics ASAP 2020+. The Density Functional Theory (DFT) model for slit shaped pores was used to calculate pore density.

e) Scanning transmission electron microscopy (S-TEM):

To prepare grids for the cross-section imaging of Fe/CMS samples, small pieces of each sample were embedded in Araldite 6005 epoxy and cured at 60°C for 48 hours before cutting with a diamond knife using a Leica UC7 ultramicrotome. The samples were cut at a thickness of 100nm. The microtomed slices were collected and deposited onto continuous carbon grids for (S)TEM analysis. High Angle Annular Dark Field (HAADF)-STEM images were obtained on a Nion

UltraSTEM100 operating at 100kV using a 30mrad probe. ImageJ software was used to perform particle size analysis. While the TEM images shown in Figure 8 are representative, several images from locations across the prepared TEM grid were used to conduct particle size analysis.

f) Raman spectroscopy:

Raman spectroscopy was performed on the CMS membrane samples using a Renishaw InVia Raman Microscope equipped with a 532 nm excitation wavelength at 10 mW power.

g) H_2 chemisorption:

H_2 static chemisorption experiments were performed using Micromeritics ASAP 2020 instrument. Approximately 0.08 g of the Fe/CMS sample was reduced under H_2 (industrial, Airgas) flow held at 500 ˚C (ramp rate of 10.0 ˚C/min) for 120 min and then evacuated for 240 min at 500 ˚C. The tube was cooled to 35 ˚C (cooling rate of 10.0 ˚C/min) followed by evacuation for 240 min. The adsorption of H_2 on Fe was performed at 100 ˚C.[4,5] H_2 gas (Airgas) was dosed at each pressure point (pressure ranges from 70 mmHg to 450 mmHg), and the quantity of H_2 absorbed was monitored by a TCD. The isotherms from the first and repeat analyses were plotted, and the linear region between 200 – 350 mmHg (200, 250, 300, and 350 mmHg) was used to calculate the difference in adsorbed quantity from the intercepts of each isotherm. This intercept difference (Δq_{chem}, μmol H_2/g_{cat}) isolates the irreversibly chemisorbed H_2 on metallic Fe^0 from weakly bound physisorbed H_2. A dissociative stoichiometry of $H_2:Fe_{surface}$ = 1:2 was assumed in calculating the surface site density. Reproducibility was evaluated by performing duplicate H_2 chemisorption measurements for each sample using the same pretreatment and adsorption procedure. The corresponding Fe surface-site densities were reported as the mean ± standard deviation (n=2), and the relative standard deviation was used as an additional measure of

experimental variability. The average Fe surface site density obtained from the duplicate measurements was subsequently used to calculate the turnover frequency (TOF) for each catalyst. The calculated site densities represent H_2-accessible Fe surface sites generated under the specific pretreatment conditions. Because H_2 uptake can depend on the oxidation state, reducibility, and accessibility of the Fe species, differences in these properties among samples may influence the measured chemisorption capacity and, consequently, the calculated site densities and TOF values.

<u>Separation properties measurements:</u>

Table S2 Comparison of NH_3 separation performance across reported membrane materials.

Membranes	NH_3 Permeability (Barrer)	Temperature (°C)	Reference
Aquivion C87-05	6690	35	[6]
ZIF-21	5	25	[7]
MFI	6870	50	[8]
TEOS	0.152 (mixed gas)	50	[9]
Metal_free CMS	1778	35	This work
Fe_dp/CMS	261	35	This work
Fe_cc/CMS (high Fe loading)	946	35	This work
Fe_cc/CMS_1 (low Fe loading)	8237	35	This work

Table S3. NH_3 Permeation and diffusion coefficients for different CMS membranes at 35 ˚ C and 30 psi.

CMS membrane type	Replicates	Permeation (Barrer)	Diffusion coefficients ($\frac{cm^2}{s}$)
**Metal_Free CMS**	Rep 1	1191.1	7.9×10^{-8}
	Rep 2	1481.3	1.3×10^{-7}
	Rep 3	1778.4	1.0×10^{-7}
**Fe_dp/ CMS**	Rep 1	209.7	1.6×10^{-8}
	Rep 2	260.6	2.1×10^{-8}
	Rep 3	135	9.5×10^{-9}
**Fe_cc/ CMS**	Rep 1	198.2	1.6×10^{-8}
	Rep 2	945.8	7.3×10^{-8}
	Rep 3	210.1	1.6×10^{-8}
**Fe_cc/ CMS_1**	Rep 1	8236.5	5.0×10^{-7}
	Rep 2	401.3	2.8×10^{-8}
	Rep 3	4817.1	3.1×10^{-7}
	Rep 4	4874.4	3.5×10^{-7}

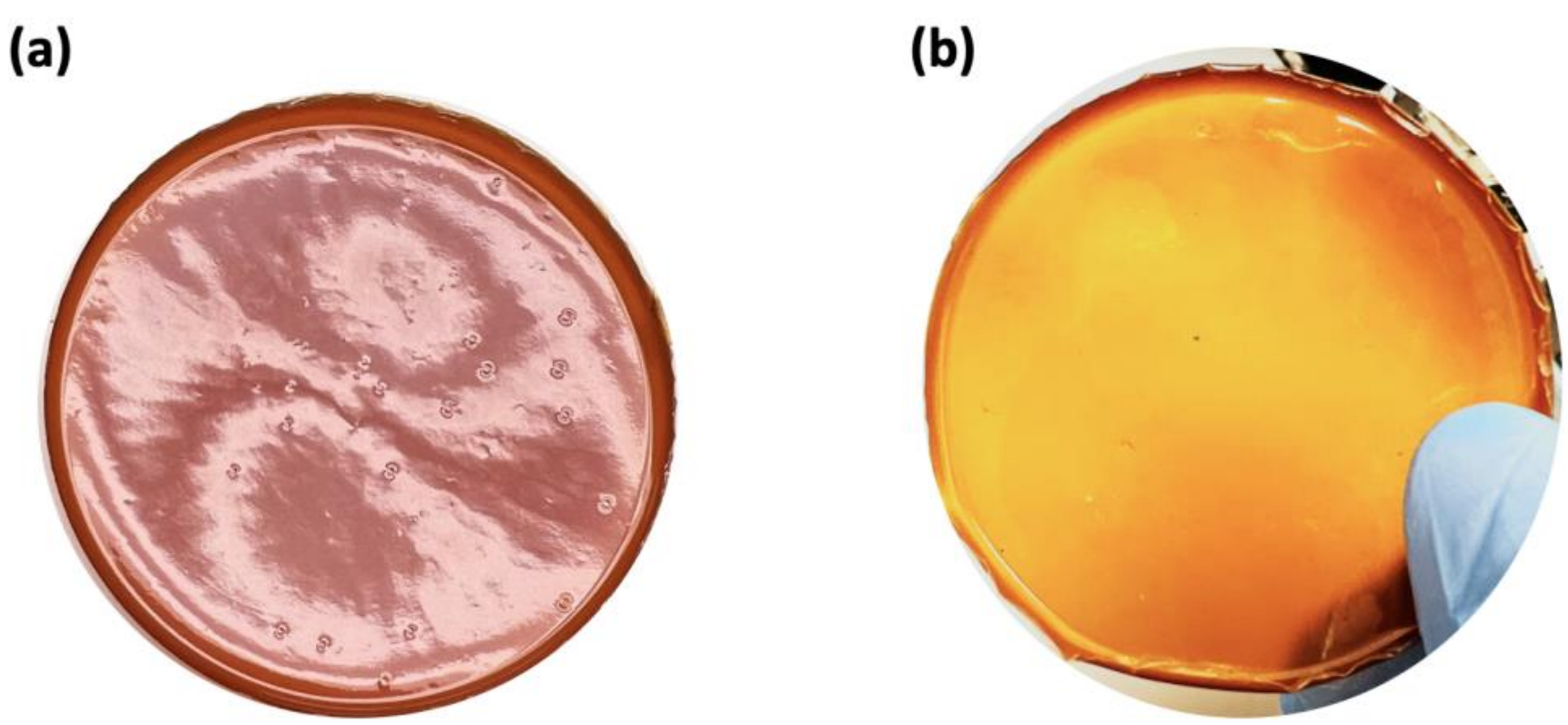

Figure S4. (a) (0.070g of $Fe(acac)_2$) Fe_cc polymer permeation sample, (b) (0.030g of $Fe(acac)_2$) Fe_cc polymer permeation sample.

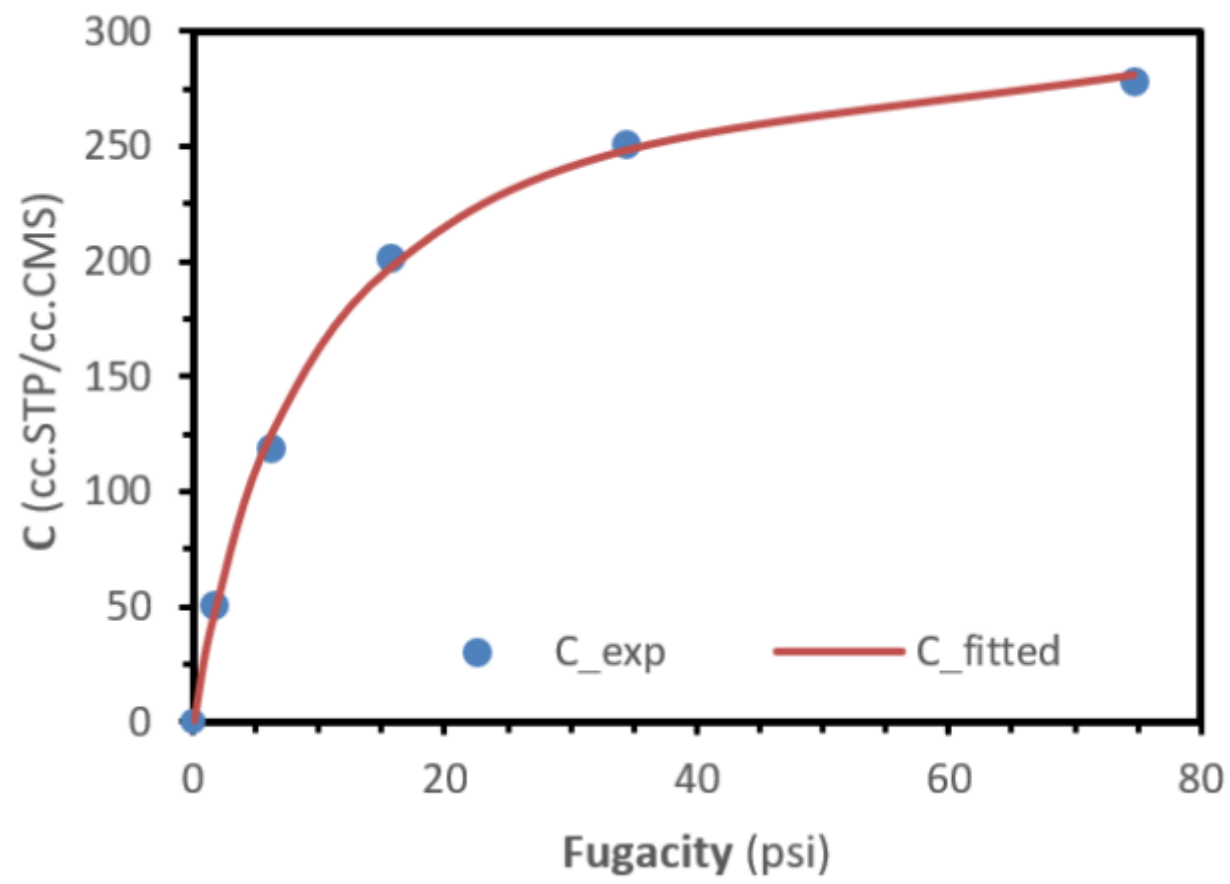

Figure S5. NH_3 sorption isotherm regression at 35 ˚ C for metal_free CMS

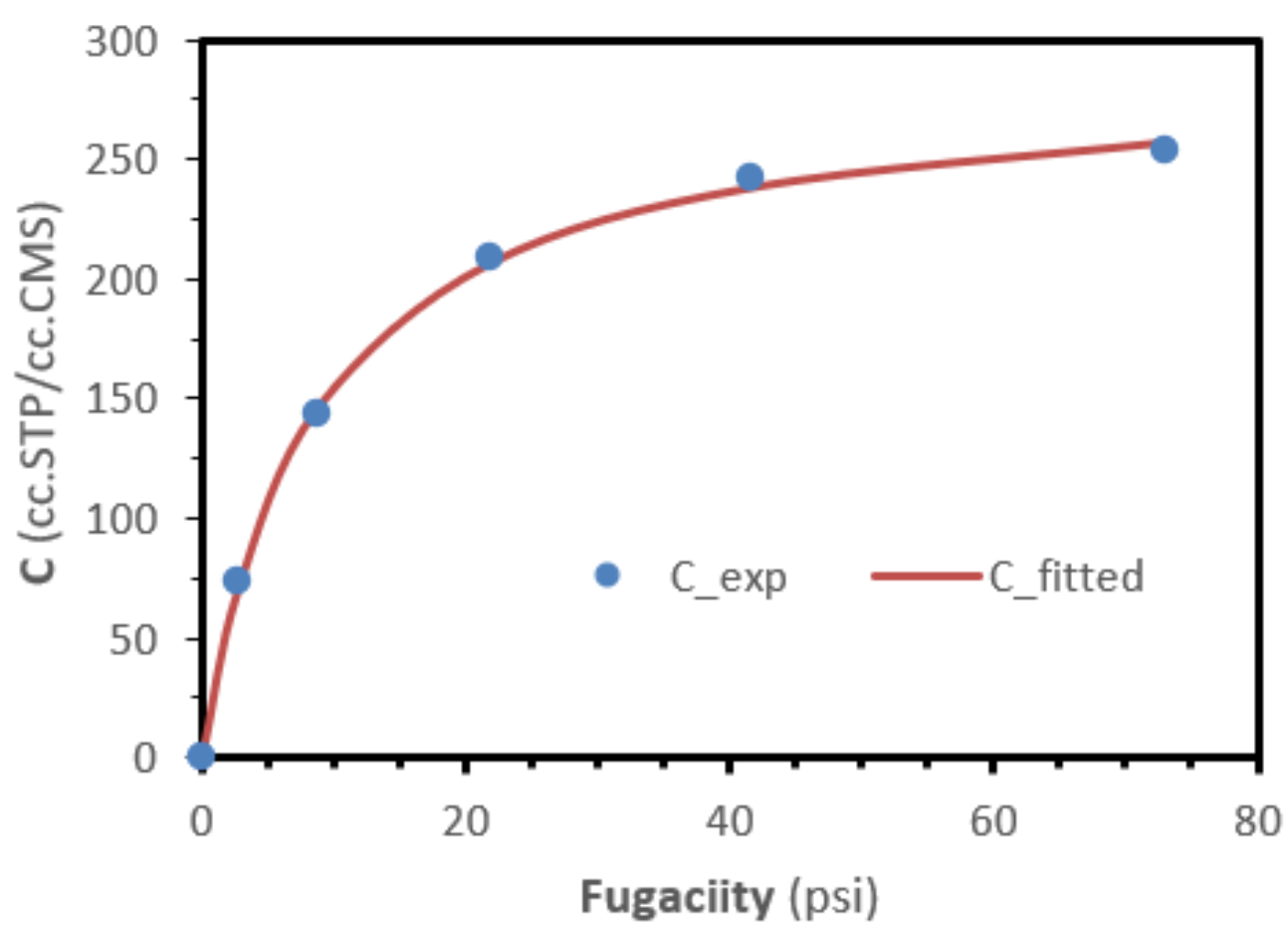

Figure S6. NH_3 sorption isotherm regression at 35 ˚C for Fe_dp/ CMS

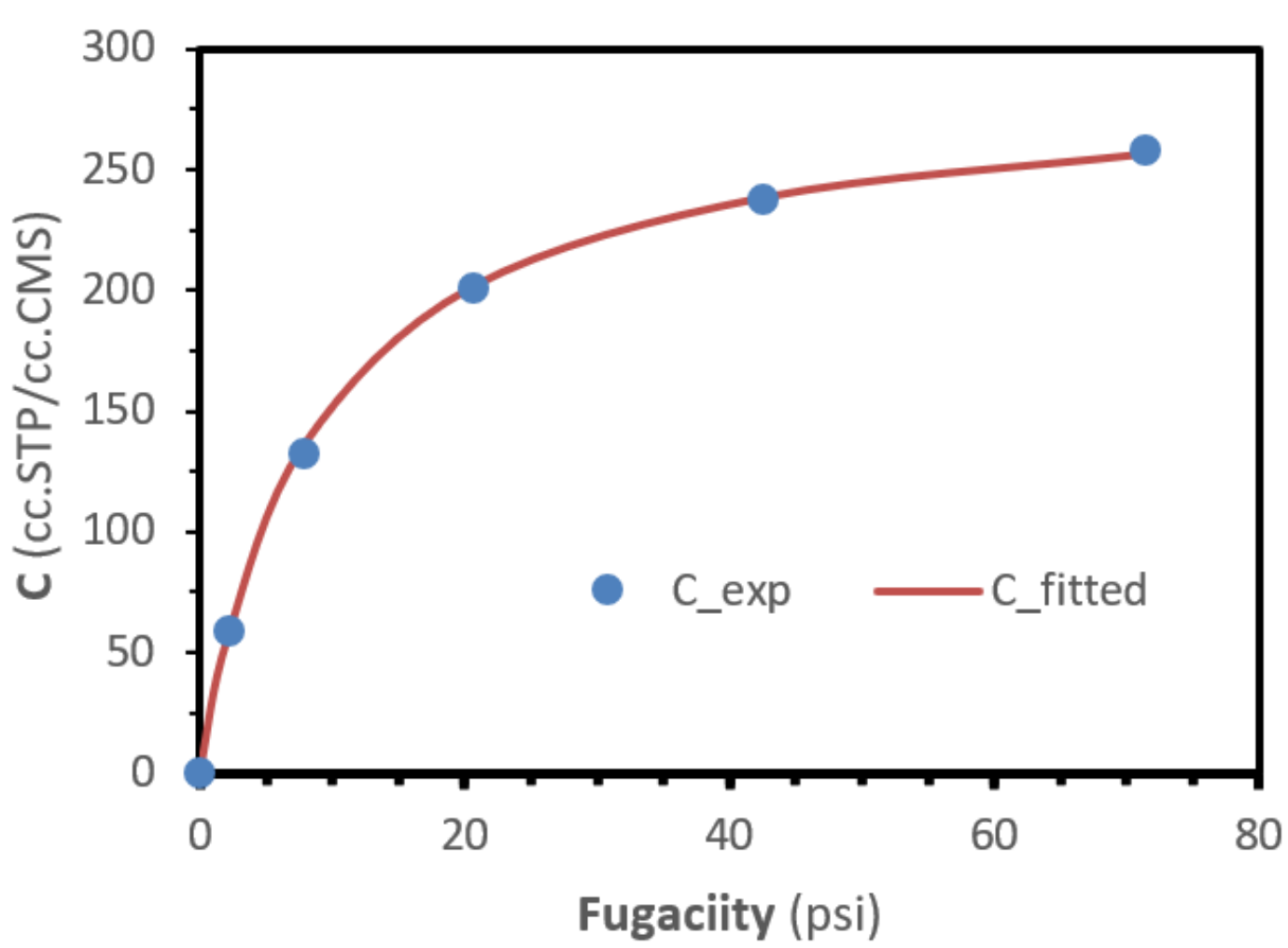

Figure S7. NH_3 sorption isotherm regression at 35 ˚C for Fe_cc/ CMS

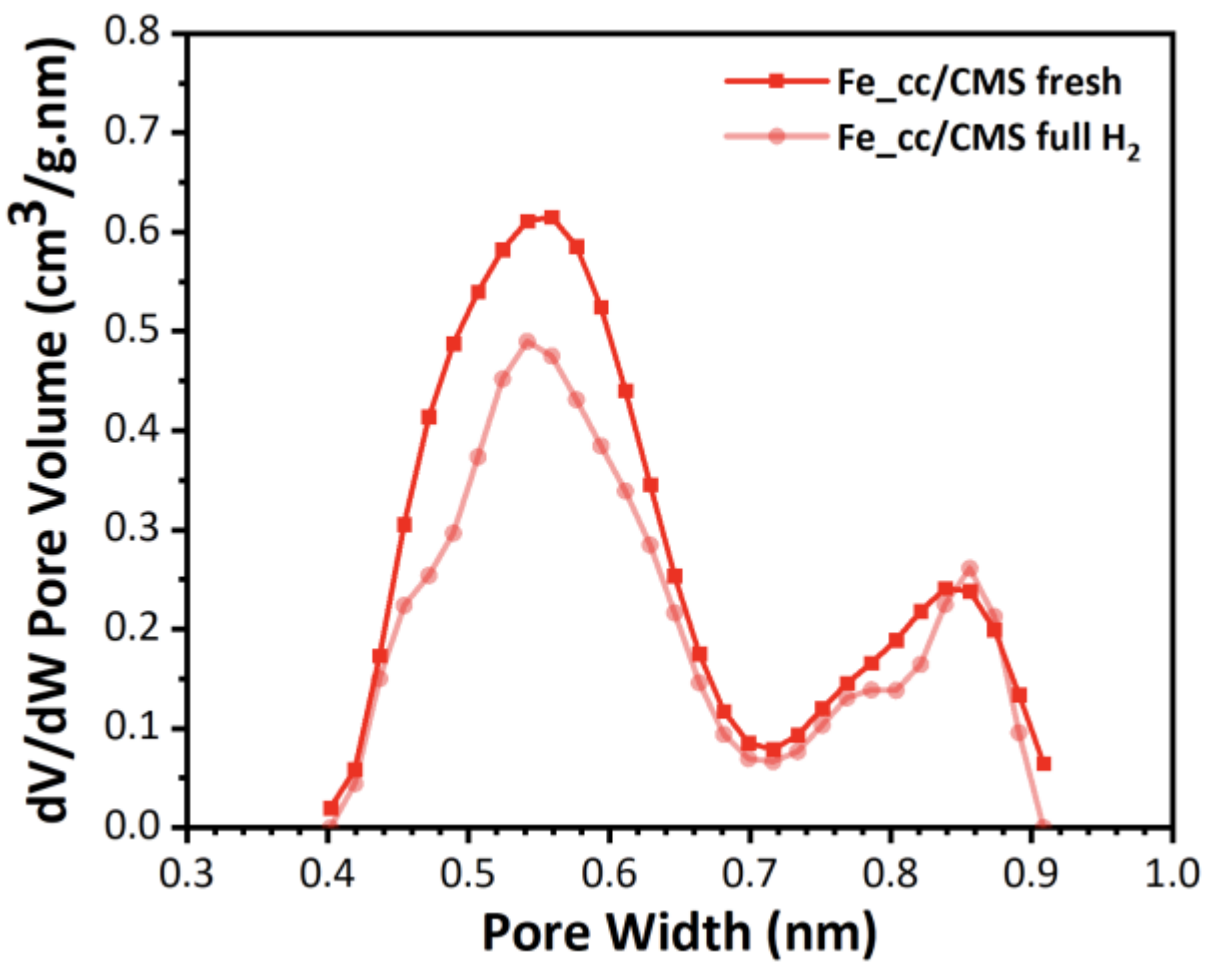

Figure S8. Pore size distributions obtained from CO_2 physisorption at 0 ˚C for Fe_cc/CMS fresh and Fe_cc/CMS full H_2 exposure samples

Reactivity experiments:

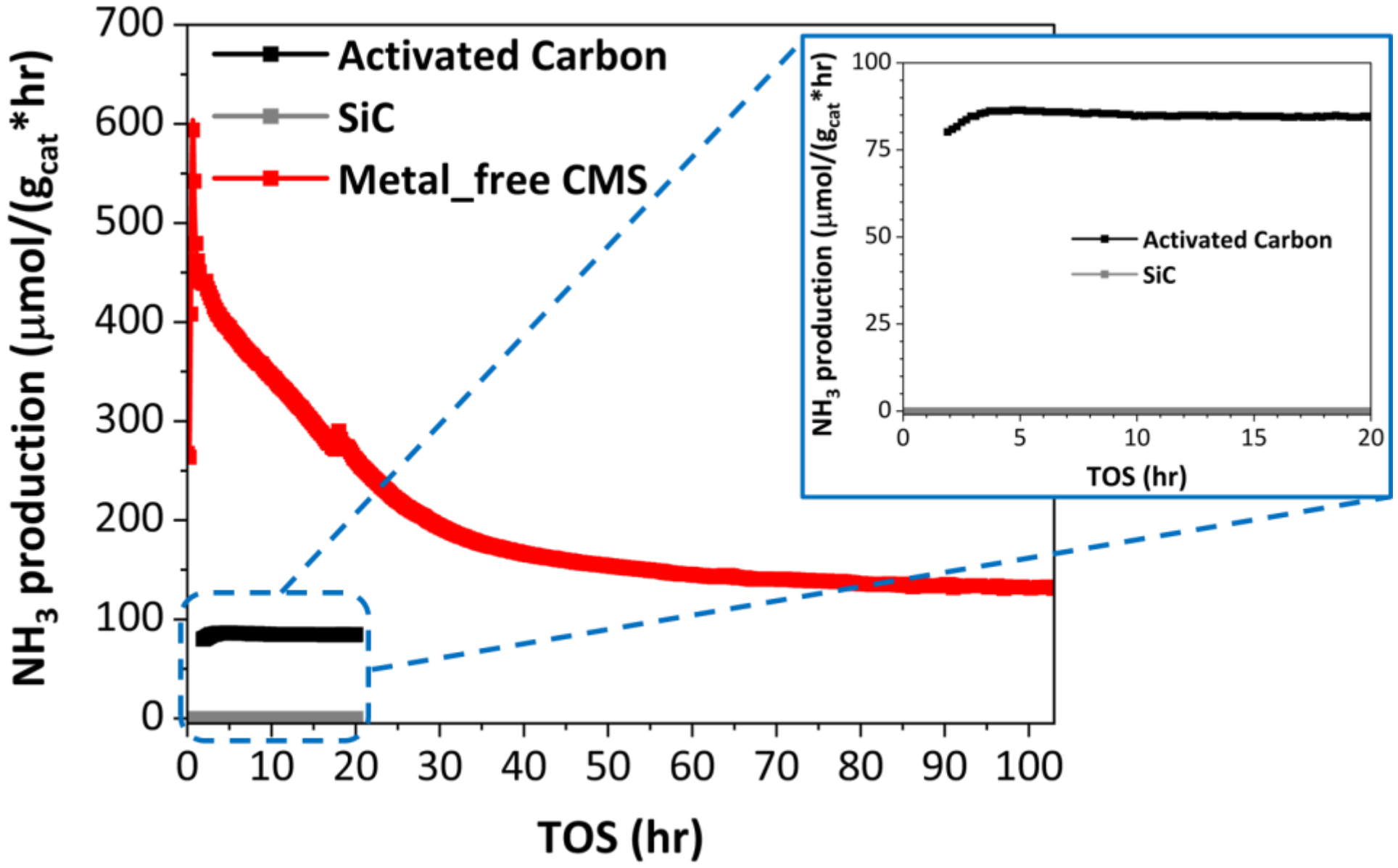

Figure S9. NH_3 production over Activated carbon, silicon carbide, and metal-free CMS. Reaction conditions: 500 °C, 400 psi, Total flow: 30 ml/min (3:1 H_2:N_2)

Table S4. Comparison of NH_3 production rates for conventional catalyst and CMS membrane samples.

	**Average NH_3 production at steady state**	
	**Per sample loading, µmol/(g_{cat}*hr)**	**Per metal loading, µmol/(g_{Fe}*min)**
Fe_dp/CMS	750	4370
Fe_cc/CMS	728	1207
Fe_IWI/CMS	-	-
Metal-free CMS	-	-
Fe/C	1329	4000

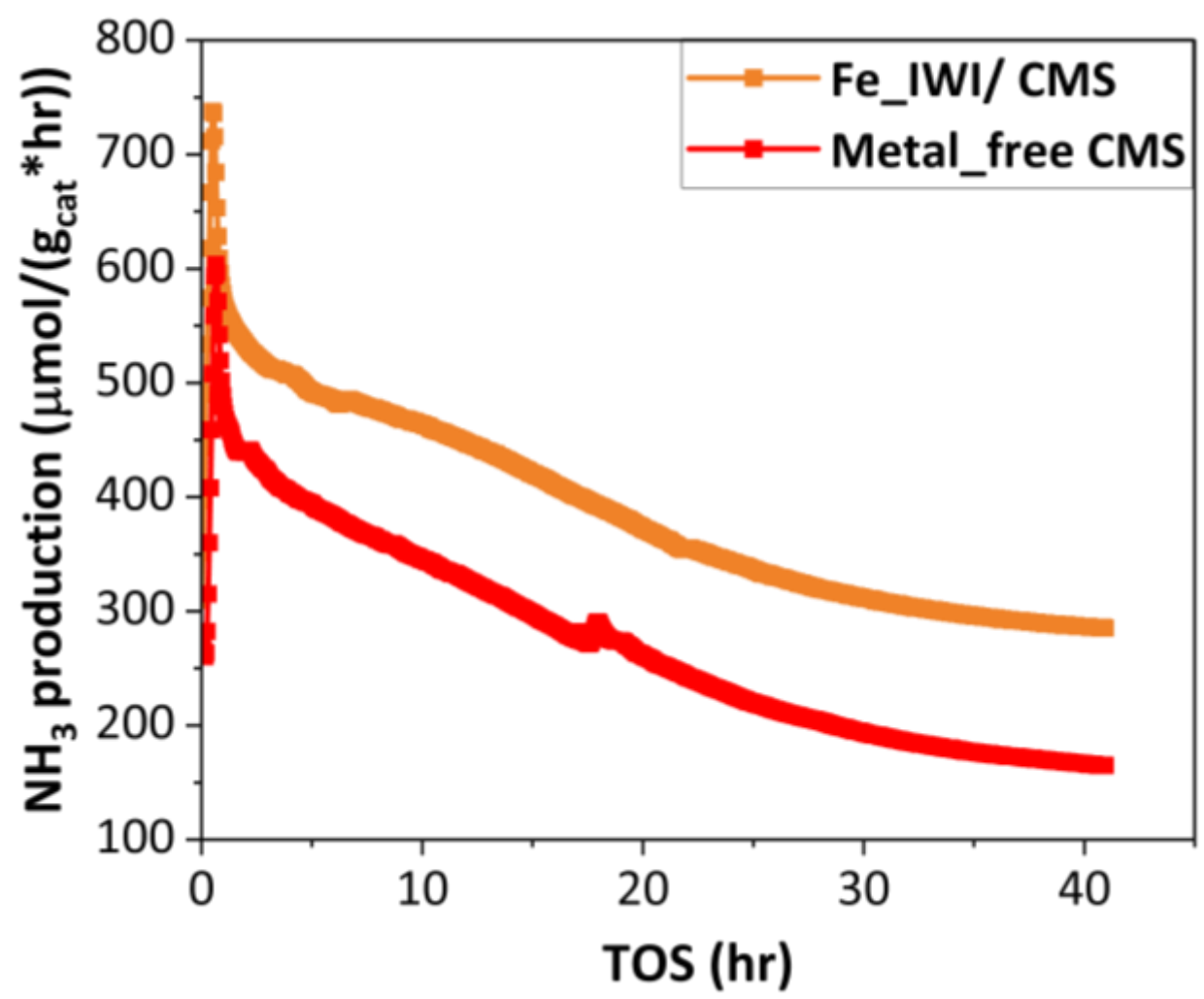

Figure S10. NH_3 production rates are normalized by weight of catalyst loading of Fe_IWI/ CMS and metal-free CMS. Reaction conditions: 500 °C, 400 psi (27.58 bar), Total flow: 30 ml/min (3:1 H_2:N_2)

Table S5: Fe loaded in CMS membrane and traditional catalyst samples.

Samples (full name)	Samples Abbreviation	Fe wt.% in sample
Fe co-casting (2 – 3 wt.%) carbon molecular sieve membrane	Fe_cc/CMS	2
Fe dip-coating (2 – 3 wt.%) carbon molecular sieve membrane	Fe_dp/CMS	1
Fe Incipient Wetness Impregnation (2 – 3 wt.%) carbon molecular sieve membrane	Fe_IWP/CMS	1.23
Fe Incipient Wetness Impregnation (20 wt.%) activated carbon	Fe/C	19.1

H_2 chemisorption measured Fe-site densities:

Table S6. H_2 chemisorption measured Fe site densities and corresponding turnover frequency (TOF) of Fe/CMS used in this study

Samples	H_2-accessible Fe site density[a] (μmol/g_{cat})	Average TOF (min^{-1})
Fe_cc/CMS	4.90 ± 4.56	2.48
Fe_dp/CMS	7.85 ± 2.19	1.59
Fe_IWI/CMS	3.90 ± 1.90	1.23

[a]Values are reported as mean ± standard deviation from duplicate H_2 chemisorption measurements (n=2). TOFs were recalculated using the corresponding mean H_2-accessible Fe surface-site density.

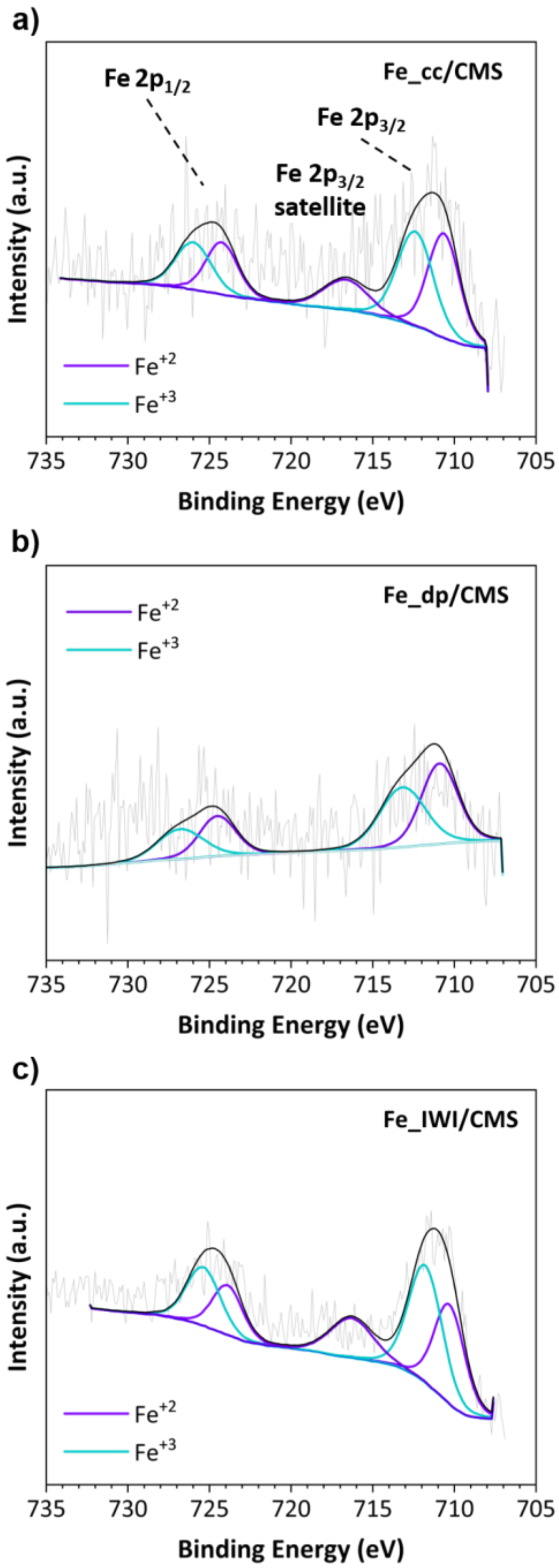

Figure S11. Deconvoluted spectra of post reaction Fe/CMS membrane materials in the Fe 2p region

__Determination of sp^3:sp^2 carbon using XPS measurements:__

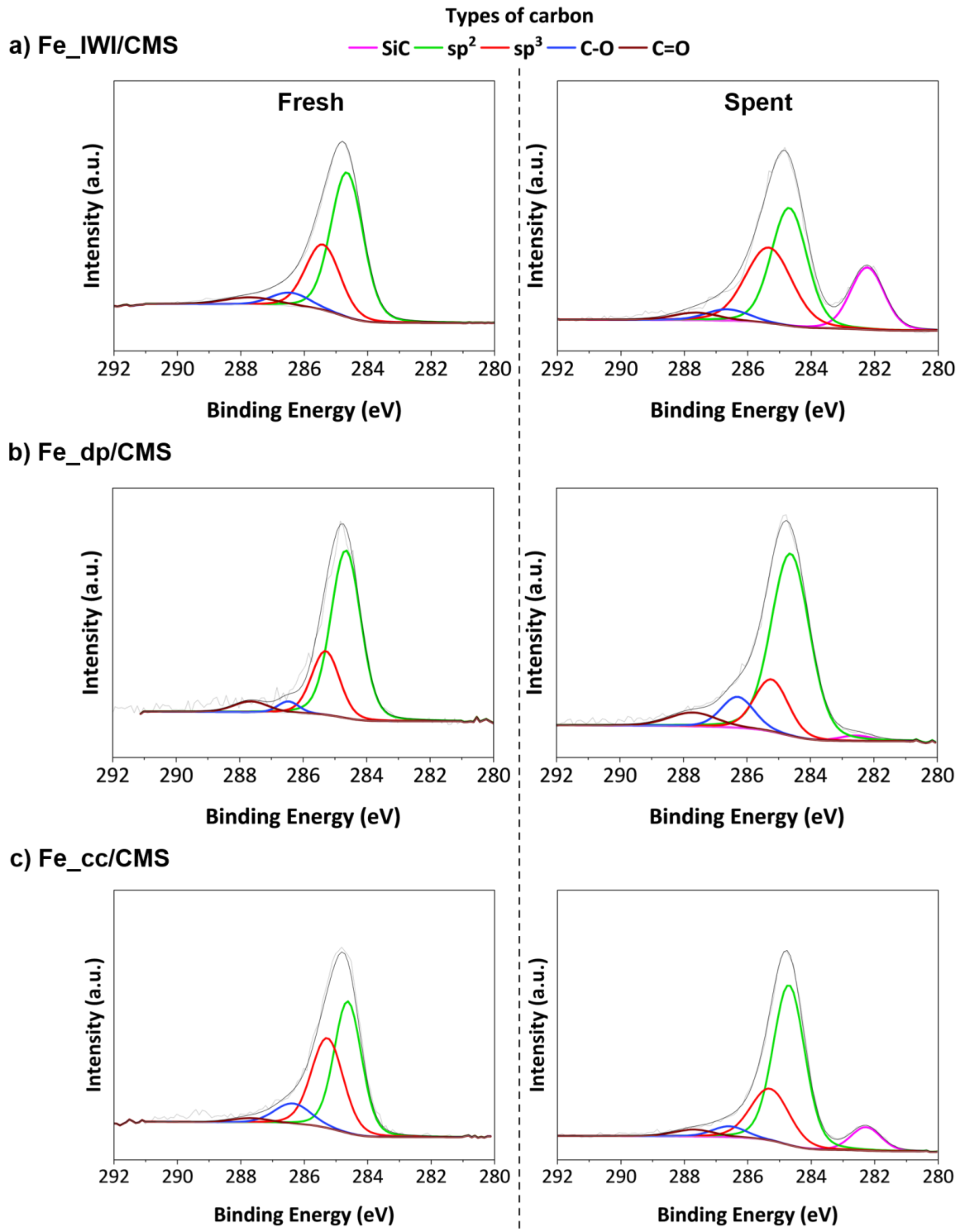

Figure S12. Deconvoluted C 1s peaks of fresh and post reaction samples of (a) Fe_IWI/CMS, (b) Fe_dp/CMS, and (c) Fe_cc/CMS

Table S7. Comparison of sp^3:sp^2 ratios calculated by deconvoluting C 1s from XPS for the samples used in this study

Samples	sp^3:sp^2 carbon	
	Fresh	Post reaction
Fe_cc/CMS	0.79	0.40
Fe_dp/CMS	0.35	0.25
Fe_IWI/CMS	0.49	0.84

<u>Particle size distribution from electron microscopy:</u>

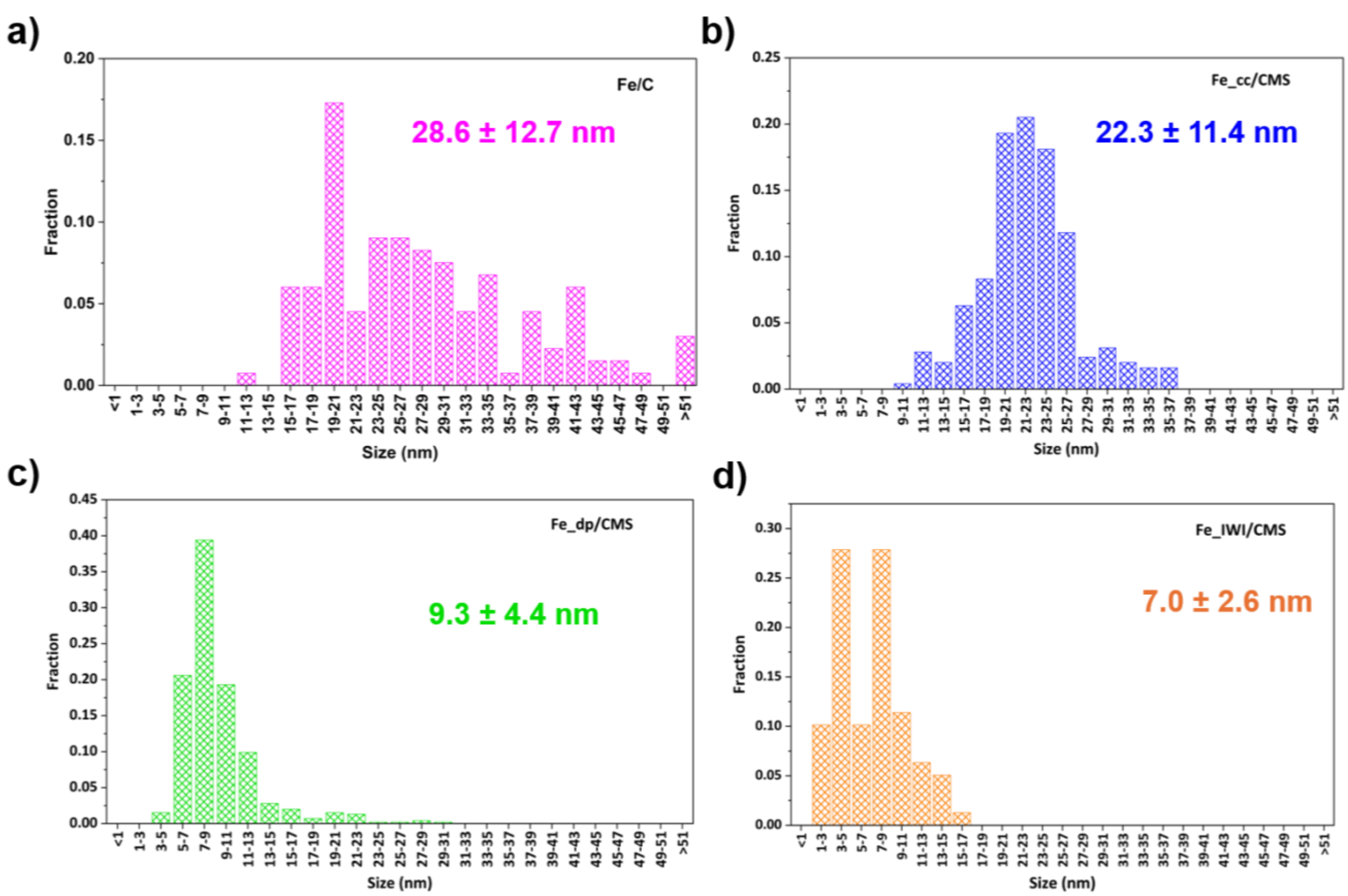

Figure S13. Particle size distribution from bright-field TEM images of (a) Fe/C and (d) Fe_IWI/CMS, and HR-TEM images of (b) Fe_cc/CMS and (c) Fe_dp/CMS

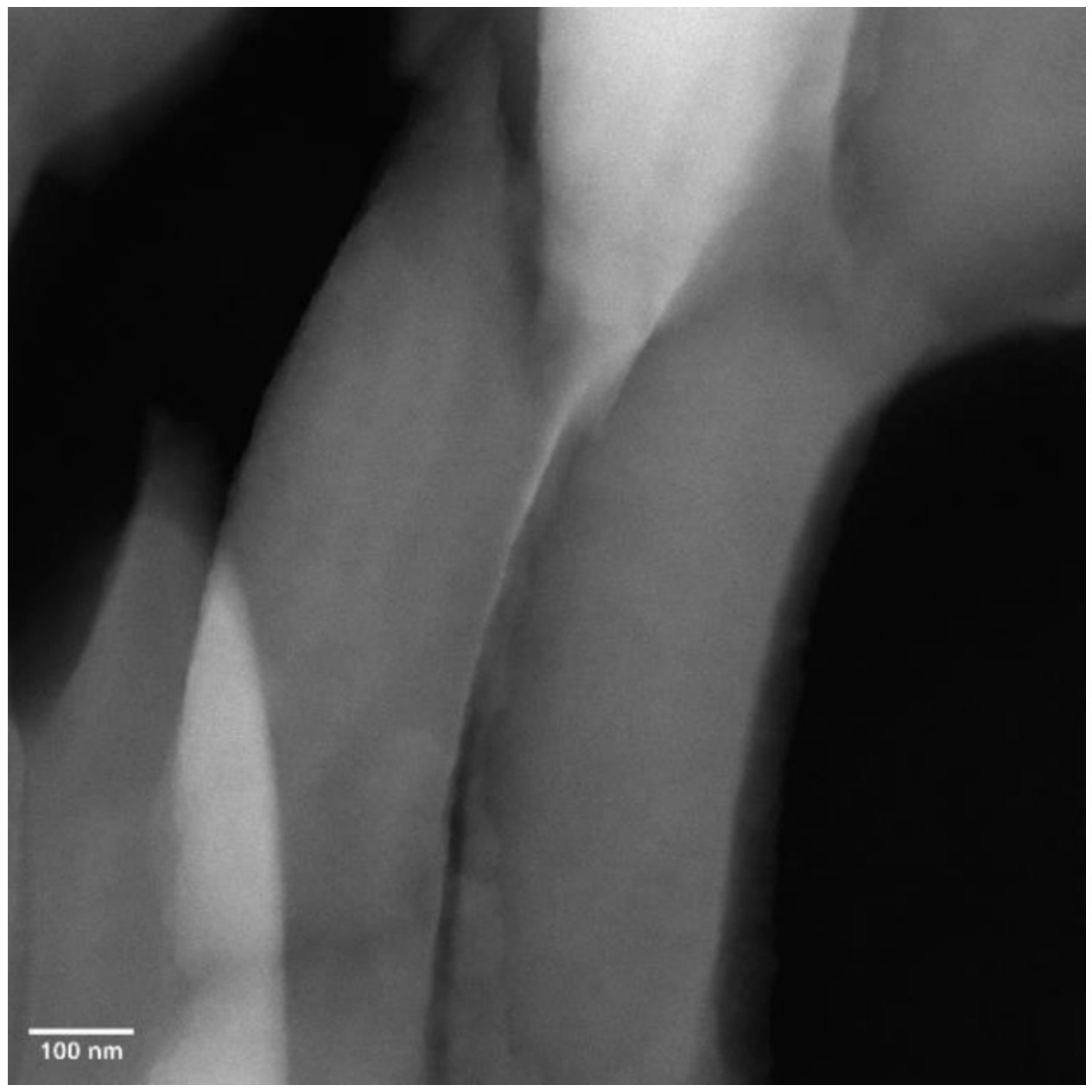

Figure S14. HR-TEM image of cross-sectional metal free CMS membrane

References:

(1) Koros, W. J.; Paul, D. R. Design Considerations for Measurement of Gas Sorption in Polymers by Pressure Decay. *J. Polym. Sci. Polym. Phys. Ed.* **1976**, *14* (10), 1903–1907. https://doi.org/10.1002/pol.1976.180141014

(2) Sanyal, O.; Hays, S. S.; León, N. E.; Guta, Y. A.; Itta, A. K.; Lively, R. P.; Koros, W. J. A Self-Consistent Model for Sorption and Transport in Polyimide-Derived Carbon Molecular Sieve Gas Separation Membranes. *Angew Chem Int Ed* **2020**, *59* (46), 20343–20347. https://doi.org/10.1002/anie.202006521

(3) Ning, X. Carbon Molecular Sieve Membranes for Nitrogen/Methane Separation. Ph.D., Georgia Institute of Technology, 2014. https://wvu.idm.oclc.org/login?url=https://www.proquest.com/dissertations-theses/carbon-molecular-sieve-membranes-nitrogen-methane/docview/3275488144/se-2?accountid=2837

(4) Venter, J. J.; Vannice, M. A. Diffuse Reflectance Infrared Fourier Transform Spectroscopic Investigation of the Decomposition of Carbon-Supported Iron Carbonyl Clusters. *J. Phys. Chem.* **1989**, *93* (10), 4158–4167. https://doi.org/10.1021/j100347a054

(5) Vannice, M. A. *Kinetics of Catalytic Reactions*; Springer US: Boston, MA, 2005. https://doi.org/10.1007/b136380

(6) Signorini, V.; Askin, A.; Oldani, C.; Minelli, M.; Giacinti Baschetti, M. Study on Ammonia Transport and Separation in Aquivion® Perfluoro Sulfonated Acid Membranes. *Journal of Membrane Science* **2024**, *697*, 122564. https://doi.org/10.1016/j.memsci.2024.122564

(7) Wei, Q.; Lucero, J. M.; Crawford, J. M.; Way, J. D.; Wolden, C. A.; Carreon, M. A. Ammonia Separation from N2 and H2 over LTA Zeolitic Imidazolate Framework Membranes. *Journal of Membrane Science* **2021**, *623*, 119078. https://doi.org/10.1016/j.memsci.2021.119078

(8) Duan, X.; Kim, D.; Narasimharao, K.; Al-Thabaiti, S.; Tsapatsis, M. High-Performance Ammonia-Selective MFI Nanosheet Membranes. *Chem. Commun.* **2021**, *57* (5), 580–582. https://doi.org/10.1039/D0CC07217F

(9) Kanezashi, M.; Yamamoto, A.; Yoshioka, T.; Tsuru, T. Characteristics of Ammonia Permeation through Porous Silica Membranes. *AIChE Journal* **2010**, *56* (5), 1204–1212. https://doi.org/10.1002/aic.12059
